# Nanoelectroanalysis with Carbon Nanopipettes Based on Prussian Blue-NiHCF for Hydrogen Peroxide Sensing

**DOI:** 10.1021/acs.analchem.6c02551

**Published:** 2026-06-25

**Authors:** Antonino Biagio Carbonaro, Gregorio Laucirica, Gastón A. Crespo, María Cuartero

**Affiliations:** † 16728 UCAM-SENS, Universidad Católica San Antonio de Murcia, UCAM HiTech, Avda. Andrés Hernández Ros, 1, Murcia 30107, Spain; ‡ Department of Chemistry, KTH Royal Institute of Technology, Teknikringen 30, Stockholm SE-114 28, Sweden

## Abstract

Carbon nanopipettes (CNPs) have emerged as powerful tools to track intracellular redox processes with high spatial resolution. Their implementation for robust H_2_O_2_ sensing, however, is still limited by stability and mechanistic constraints largely attributed to the surface chemistry of the electrode under nanoconfinement. To address this challenge, we report for the first time a combination of Prussian Blue–Nickel hexacyanoferrate (PB-NiHCF) modified carbon nanopipettes for the electrochemical detection of H_2_O_2_ under nanoconfined conditions. Electrodeposition strategies and stabilization protocols for the growth of PB-NiHCF thin films are thoroughly inspected. Cyclic voltammetry and double-potential step chronoamperometry (DPSC) are used to rationalize the thin-layer regime established within the nanometric inner tip of the CNPs. Attention is devoted to the nature and catalytic properties of the electrodeposited materials, which are key factors governing the sensing performance. Notably, both the open-circuit potential of the modified nanopipette and the management of the applied potential at the working electrode prove crucial for the detection mechanism. The analytical performance of the system is evaluated by DPSC, demonstrating stable sensing and a linear response up to 500 μM H_2_O_2_ (*R* ^2^ = 0.996), with a limit of detection of 28.9 μM at physiological pH, supporting further in vivo single-cell analysis. Overall, this work provides an analytical framework for H_2_O_2_ detection in thin-layer electrochemical cells, opening new perspectives for nanoscale electroanalysis in confined environments and coulometric sensor development.

## Graphical abstract

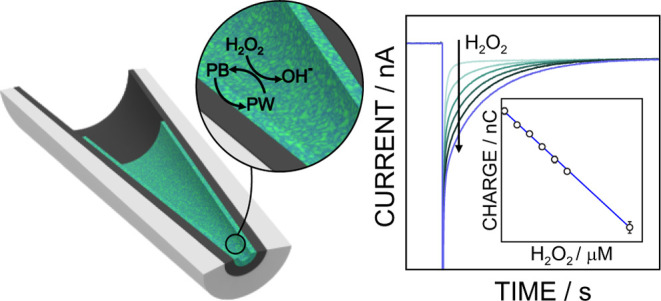

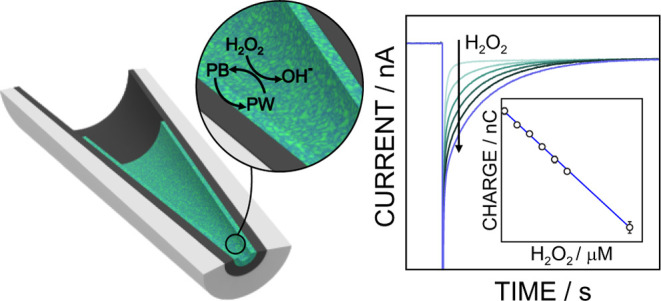

## Introduction

Electroanalysis is entering a new era driven by the challenge of investigating redox processes in highly confined and biologically relevant environments. From probing the dynamics of single living cells to monitoring electroactive species at the nanoscale, these advances demand the development of nanoelectrodes capable of operating with both high spatial resolution and adequate sensitivity.^1,2^ Carbon nanopipettes (CNPs), ultrasmall pipettes with conductive carbon-coated openings,^3,4^ have gained particular attention owing to their unique performance, spanning from fundamental physicochemical studies,^5,6^ and biological applications.^3,7^ Because of the reduced dimensions and the possibility of confining volumes from the picoliters to femtoliters, CNPs can be conceived as nanoscale working electrodes (WEs) pursuing unprecedented opportunities for electrochemical measurements.^3,8^ Moreover, CNPs are promising candidates as sensing probes to monitor biologically relevant molecules in single living cells^9,10^ and biological tissues.^11^

Depending on their pore size and inner tip, CNPs can operate under a thin-layer electrochemical regime that facilitates the complete electrolysis of confined electroactive species, thereby allowing for calibration-free sensing and coulometric measurements.^12^ In previous studies, we have demonstrated the thin-layer behavior of the bare CNPs using a combination of techniques—cyclic voltammetry (CV), electrochemical impedance spectroscopy (EIS)—and numerical simulations. Besides, we shed light on the interpretation of the interplay between ionic and electronic transport, and how the experimental conditions can affect the electrochemical output.^13,14^ In this context, the surface chemistry of the inner conductive carbon walls of the CNP tip plays a pivotal role, as it represents the recognition interface between the detection unit and the environment (sample) under investigation. Indeed, previous works claimed that chemical functionalization of the inner walls, such as carboxylation for exocytosis studies or β-cyclodextrin modification for the enantioselective detection of tryptophan in single living cells, significantly enhances CNP performance.^9,15^

Although several studies have reported chemical modifications of the inner carbon walls of CNPs,^15–18^ the overall contribution to this topic remains rather limited, particularly concerning the systematic exploration of the electrochemical implications that the corresponding surface modification may introduce. Building on this gap in the state-of-the-art, herein, we investigate the use of the inner carbon coating of CNPs as a nucleation platform for the growth of Prussian Blue (PB)/Nickel hexacyanoferrate (NiHCF) thin films intended for hydrogen peroxide (H_2_O_2_) detection in a thin-layer regime. PB is a well-established “artificial peroxidase” that promotes the catalytic reduction of hydrogen peroxide (H_2_O_2_) at low overpotentials through the reversible Fe­(III)/Fe­(II) redox couple, thereby minimizing interference from coexisting electroactive species and ensuring high selectivity in biological environments.^19^ Nevertheless, a major limitation of PB is its restricted operational stability, particularly under conditions where hydroxide ions (OH^–^) accumulate, which may lead to solubilization or degradation of the PB film.^20^ In this work, PB was combined with NiHCF to enhance the mechanical stability and durability of the PB thin film^21^ making it suitable for nanoelectroanalysis purposes.

Although it is not a free radical, H_2_O_2_ is classified among reactive oxygen species (ROS). In mammalian cells, it functions as a major redox mediator that regulates a wide range of metabolic and signaling processes, reaching a high level of complexity.^22–25^ Conventional strategies for intracellular H_2_O_2_ detection mainly rely on fluorescent or redox-sensitive probes.^26–28^ To date, only a few studies have reported CNPs as electrochemical transducers for H_2_O_2_. For instance, Wang et al. developed an H_2_O_2_-sensitive CNP based on the decoration of the inner carbon walls with metal nanoparticles. This device showed a linear response in the mM range by following H_2_O_2_ oxidation at +0.4 V by voltammetric measurements.^18^ Mirkin and coworkers introduced a MOF-based CNP in a disk-type configuration, displaying catalytic activity for the reduction of H_2_O_2_.^17^ Furthermore, Yu et al. developed a CNP functionalized with cobalt phthalocyanine, a complex that catalyzes the oxidation of H_2_O_2_ at +0.5 V vs Ag/AgCl, and performed chronoamperometric measurements directly inside HeLa cells.^16^ Also, two studies describe the use of the PB with carbon nanopipettes as an amperometric sensor for H_2_O_2_.^29,30^ Notably, immobilizing the artificial peroxidase within the CNP may give rise to a thin-layer electrochemical regime that not only enhances the attainable current magnitude but also establishes the basis for the development of calibration-free sensing platforms.^31^

In the present work, the sensing mechanism and the analytical performances of the PB/NiHCF-modified CNPs (PB-Ni-CNPs) are assessed toward H_2_O_2_ via CV and double-potential step chronoamperometry (DPSC). The synergy between these two electroanalytical techniques revealed that certain experimental parameters, such as the diffusion of the electroactive species within the CNP tip and the open-circuit potential (OCP) of the working electrode, play a crucial role in the effective detection of H_2_O_2_. This is mainly driven by the chemical composition of the PB/NiHCF-coated WEs. Overall, we aim to innovate on the methodological understanding of H_2_O_2_ detection under thin-layer conditions, emphasizing the control of critical parameters governing confined electrochemical measurements with PBs. Moreover, to the best of our knowledge, this represents the first report on the H_2_O_2_ detection with PB-Ni-CNPs under a thin-layer regime, providing a methodological framework for future reliable, quantitative measurements in highly confined electrochemical environments.

## Experimental Section

### Materials

Potassium chloride (KCl, 99.5–101.0%), potassium hexacyanoferrate (III) (K_3_[Fe^(III)^(CN)_6_], >99.0%), hydrogen peroxide (H_2_O_2_, 30%), l-Ascorbic Acid (C_6_H_8_O_6_, 99.0–100.5%), dipotassium hydrogen phosphate (K_2_HPO_4_, 99.2%), potassium dihydrogen phosphate (KH_2_PO_4_, 99.6%), and hydrochloric acid (HCl, 1N) were purchased from VWR. d-(+)-Glucose anhydrous (C_6_H_12_O_6_, 99.0%) and uric acid (C_5_H_4_N_4_O_3_, 99.0%) were provided by Thermo Scientific. Nickel­(II) chloride hexahydrate (NiCl_2_·6H_2_O, 99.9%) and iron­(III) chloride (FeCl_3_, ≥97.0%) were purchased from Sigma-Aldrich. Quartz capillary tubes without filament (0.7 mm inner diameter, 1.0 mm outer diameter, and 10 cm length) were obtained from Sutter Instrument (Novato, CA). All the chemicals were used as received. All solutions were prepared in ultrapure water (18.2 MΩ cm at 25 °C, Milli-Q water system, Merck Millipore).

### Fabrication of the Carbon Nanopipettes and Their Modification

Quartz nanopipettes were first fabricated from quartz capillaries employing the laser pulling technique using a CO_2_ laser-based puller, P-2000 (Sutter Instrument), according to the following parameters: HEAT = 700; FILAMENT = 4; VELOCITY = 60; DELAY = 145; PULL = 175. To obtain the CNPs, an amorphous carbon film was deposited on the inner walls by atmospheric pressure chemical vapor deposition (APCVD) using methane (CH_4_) as the carbon source (925 °C; CH_4_/Ar flow rates 0.2/0.6 L min^–1^, 3.5 min). PB thin films were then electrodeposited inside the inner walls of the CNPs. The PB precursor solution was prepared by adding 500 μL of 5 mM FeCl_3_ in 0.2 M HCl dropwise into 500 μL of 5 mM K_3_[Fe­(CN)_6_] in 0.2 M KCl. The CNP tip was immersed in the resulting mixture for 60 s to allow precursor cocktail filling. Electrodeposition was then carried out by three CV scans between +1.0 V and −0.2 V at 50 mV s^–1^, using 50 mM KH_2_PO_4_ in 0.3 M KCl, pH 4.3, as the supporting electrolyte. This step was followed by five sets of 20 CV scans in the potential range +1.0 V to −0.4 V at 100 mV s^–1^. This last stage was performed to improve the PB structural stability due to K^+^ cations incorporation in the lattice crystal vacancies.^19,29,32^ The last step was the creation of the NiHCF overlayer via electrodeposition.^21^ For this, 500 μL of 2 mM NiCl_2_·6H_2_O in 0.016 M HCl/0.8 M KCl were added dropwise into 500 μL of 1 mM K_3_[Fe­(CN)_6_] in 0.016 M HCl/0.8 M KCl, resulting in the NiHCF precursor solution. Then, the CNP tip was immersed for 120 s in the resulting precursor, being subjected to three CV sweeps between +1.0 V and −0.2 V at 50 mV s^–1^ in the KH_2_PO_4_/KCl supporting electrolyte.

### Detection of Hydrogen Peroxide

The mechanism underlying H_2_O_2_ detection was investigated by CV and DPSC. DPSC measurements were carried out after successive fillings of the CNP tip in standard H_2_O_2_ solutions, maintaining the tip potential at +400 mV. Subsequently, a potential of +400 mV was applied as an equilibration step, followed by −200 mV for 150 s to trigger H_2_O_2_ reduction. All the potentials were applied versus a homemade Ag/AgCl wire reference electrode (RE). The charge values (*Q*) were determined from the DPSC curves by integrating the reduction peak, using the median value of the steady-state current after H_2_O_2_ reduction as the baseline. All the electrochemical characterization experiments were performed using the same CNP to minimize interbatch variability. Thus, similar behavior was consistently observed across triplicate measurements. All the electrochemical measurements were performed in 50 mM KH_2_PO_4_ prepared in 0.3 M KCl (pH 4.3) as the supporting electrolyte. Also, the device was evaluated under physiological conditions, using a 50 mM phosphate buffer system (KH_2_PO_4_/K_2_HPO_4_) prepared in 0.3 M KCl and adjusted to pH 7.4. The volume of liquid introduced into the nanopipette was accurately regulated using a pressure controller interfaced with the HEKA probe.

### Electrochemical Setup

Electrochemical depositions and stabilization steps were performed in a three-electrode configuration consisting of a platinum wire as the counter electrode (CE) and a homemade Ag/AgCl wire as the RE. The inner part of the CNPs is used as the WE, creating an electrical connection to the potentiostat by inserting an Ag wire from the back of the quartz capillary. The three electrodes were connected to the potentiostat VIONIC (Metrohm Nordic AB, Stockholm) operated with the Intello 1.6 software. The experiments were carried out inside a Faraday cage (Rittal, GmbH & Co. KG). For the investigation of the electrochemical performance toward H_2_O_2_, experiments were performed with a double patch clamp EPC 10 USB amplifier (HEKA Elektronik) coupled to a Ti2-E inverted research microscope (Nikon). The CNP sample was connected to the amplifier through a HEKA EPC probe by inserting an Ag wire from the back of the pipet. The probe position was controlled by the uMp micromanipulation system pack (Sensapex), with a 47° inclination angle relative to the solution under examination. The volume inside the CNPs was managed by a pressure controller (Sensapex, from −70 to +70 kPa) connected to the HEKA EPC probe. All the experiments were performed inside a Faraday cage (TMC).

## Results and Discussion

### Investigation of the Electrodeposition of PB-NiHCF on the Inner Walls of CNPs

All the steps included in the device preparation are illustrated in Figure 1. Electrodeposition of PB was carried out following an adjusted version of an already reported protocol.^29^ It was conducted in the supporting electrolyte solution (KH_2_PO_4_/KCl) at pH 4.3. The selection of an acidic environment was a key aspect, given the poor stability of the PB at pH values exceeding 6.4.^33^ After filling the CNP tip with the PB precursor solution, three CV scans were performed in the potential window from +1.0 V to −0.2 V at a scan rate of 50 mV s^–1^. The results are provided in Figure 2a.

**Figure 1:**
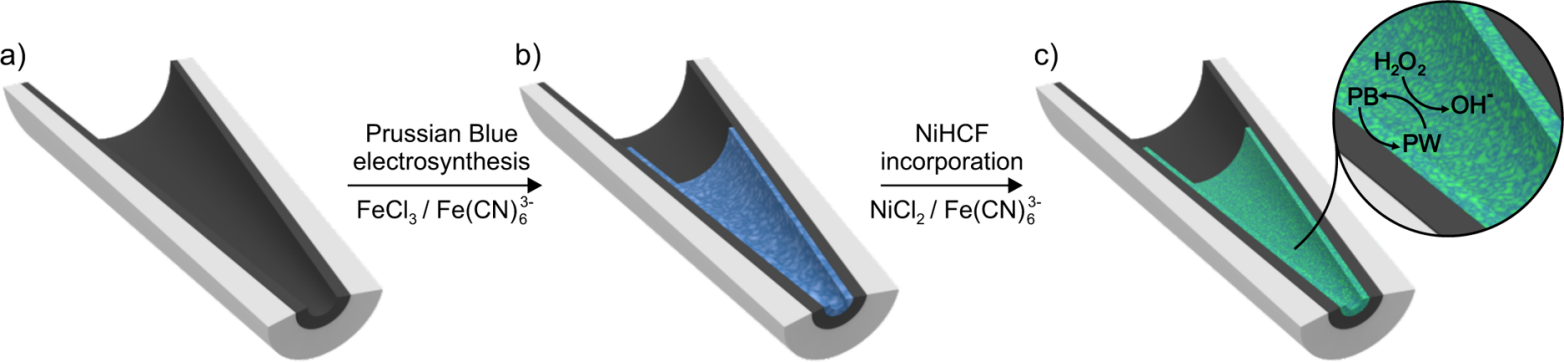
Schematic representation of the CNP fabrication. a) Bare carbon nanopipette. b) Prussian blue electrodeposition. c) Nickel hexacyanoferrate electrodeposition.

**Figure 2:**
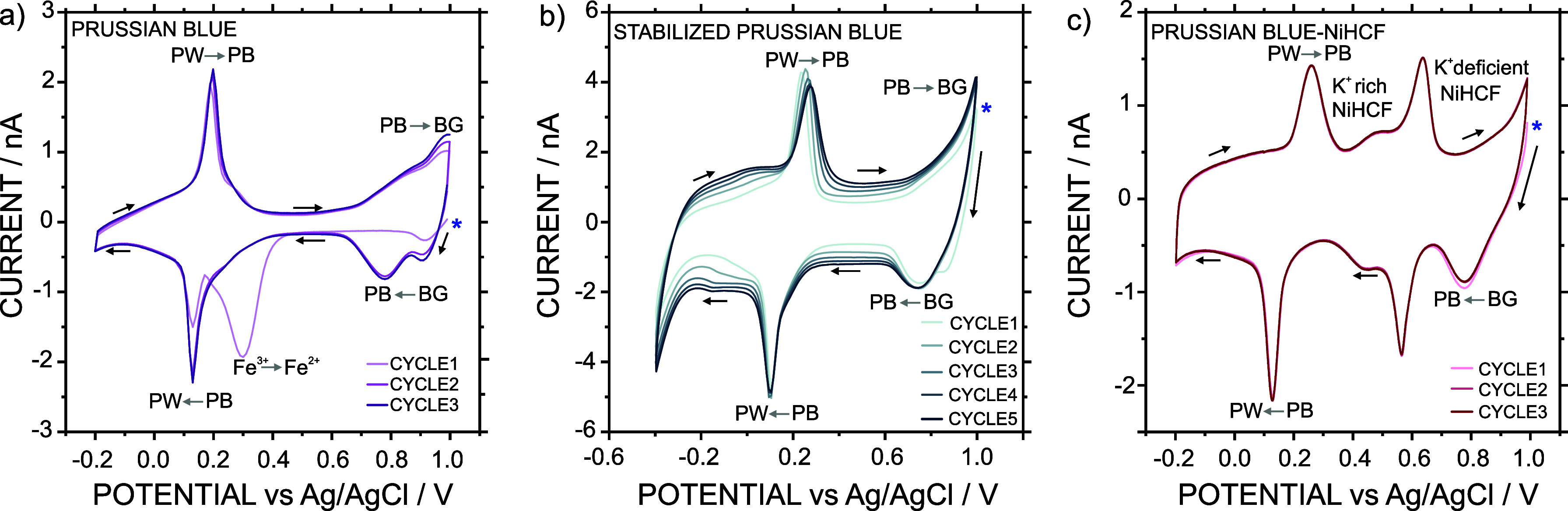
a) CVs observed in the PB electrodeposition in the CNP tip performed by three CV scans at a scan rate of 50 mV s^–1^. Initial potential: +1.0 V, forward scan from +1.0 V to −0.2 V, turning potential −0.2 V, backward scan from −0.2 V to +1.0 V, end potential +1.0 V. b) CVs observed in the stabilization of the PB-CNPs, scan rate of 100 mV s^–1^. Initial potential: +1.0 V, forward scan from +1.0 V to −0.4 V, turning potential −0.4 V, backward scan from −0.4 V to +1.0 V, end potential +1.0 V. c) CVs observed in the Nickel hexacyanoferrate (NiHCF) electrodeposition in the PB-CNPs tip performed by three CV scans at a scan rate of 50 mV s^–1^. Initial potential: + 1.0 V, forward scan from +1.0 V to −0.2 V, turning potential −0.2 V, backward scan from −0.2 V to +1.0 V, end potential +1.0 V. In all the figures, the asterisk denotes the starting potential of the CV experiment, whereas the arrow indicates the scan direction.

The first cycle shows three main cathodic features, located at +0.9 V, +0.3 V, and +0.13 V. The weak plateau at +0.9 V can be attributed to the reduction of the one-to-one Fe^III^[Fe^III^(CN)_6_] complex already present in the precursor mixture, which triggers the initial PB nucleation.^34^ Proceeding cathodically, the peak at +0.3 V was associated with the further growth and stabilization of the PB polycrystalline network through the electrochemical reduction of the Fe^III^-containing precursor species present in the complex mixture. This signal was only observed during the first cycle, confirming that the electrodeposition occurs mainly during the initial scan, due to the total consumption of the precursors. The subsequent peak at +0.13 V corresponds to the reduction of PB to its fully reduced form, denoted as Prussian White (PW).^34^ In the anodic sweep, a peak is observed at +0.18 V, corresponding to the oxidation of PW back to PB. Further oxidation continues up to +0.8 V, where PB is converted into the fully oxidized state Berlin Green (BG).^34^ From the second cycle onward, the CVs overlap almost completely, displaying a new redox process in the anodic region around +0.8 V, which can be attributed to the BG → PB transition.^19^ Finally, the PW-related redox peaks become progressively more defined and reach a constant current after the second cycle. In contrast to electrodepositions at macroelectrodes exposed to the bulk solution, where the current increases cycle by cycle due to continuous reagent supply on the WE,^35^ the current magnitude in our experiments stabilized after the second scan. This phenomenon, resulting from the immediate consumption of the confined reagents within the CNP tip, is a clear signature of the thin-layer regime.

A 1:1 molar ratio between FeCl_3_ and K_3_[Fe­(CN)_6_] was employed during PB electrosynthesis, yielding relatively narrow PB/PW redox peaks during the initial electrodeposition step (Figure 2a). Since the sharpness of the PB/PW redox peaks has been reported as an indicator of the quality of Prussian Blue layers and has been associated with a regular PB structure exhibiting homogeneous charge and ion-transfer distribution throughout the film, the observed peak profile suggests the formation of a relatively regular and homogeneous PB framework.^34^

To enhance the stability of the PB film, five sets of 20 CV scans in the range from +1.0 to −0.4 V at 100 mV s^–1^ were applied to incorporate K^+^ ions from the solution bulk into the lattice, where they acted as pillars reinforcing the framework;^29,32^ the resulting CVs after each stage are presented in Figure 2b. An increase in the capacitance and a slight peak separation were observed upon cycling, which was attributed to structural hydration leading to higher electrical resistance. Also, the progressive broadening of the anodic feature may be related to K^+^ transport within the PB lattice during the PW → PB transition. It has been previously reported that, although PB-modified electrodes exhibit excellent performance as H_2_O_2_ transducers, their stability is often limited by intrinsic structural defects, particularly for the presence of Fe­(CN)_6_ vacancies in the PB lattice.^36^ Moreover, the three-dimensional structure of PB undergoes complete hydrolysis in neutral or mildly basic environments, thereby limiting its applicability under physiological conditions.^21,37^ A strategy to overcome this drawback is the incorporation of electrochemically inert ions or the formation of core–shell architectures, where the PB is coated with a more stable analog.^21,38^ Among these, NiHCF has attracted considerable attention due to its ultrahigh cycling stability.^21,34,39^ Accordingly, the PB layer was further stabilized by electrodepositing an additional NiHCF film. After filling the PB-CNP tip with the NiHCF precursor solution, the electrodeposition was achieved by applying three CV scans in the potential range from +1.0 V to −0.2 V at a scan rate of 50 mV s^–1^. The corresponding voltammograms are shown in Figure 2c. In addition to the PB signature, two redox peaks having anodic peak potential located at +0.47 V and +0.65 V appeared, which are attributed to two different forms of NiHCF: the positive one known as potassium-rich (K_2_Ni­[Fe­(CN)_6_]) and the negative one, denoted as potassium-deficient (KNi_1,5_[Fe­(CN)_6_]).^40^ The resulting modified nanopipette, labeled as PB-Ni CNP, showed improved operational stability in physiological medium at pH 7.4 compared to the PB-only CNP (see Figure S1), demonstrating the ability of the NiHCF framework to reinforce PB thin films. As observed in Figure S2, minor variations in the electrochemical behavior between independently fabricated PB-NiHCF modified CNPs were observed during the electrodeposition and stabilization steps, mainly affecting the peak current intensities. Such behavior is likely attributed to slight differences in precursor filling inside the CNP nanotip. Nevertheless, the fabrication process of CNPs, particularly capillary pulling and carbon deposition by CVD, constitutes an intrinsic technological source of variability in nanoscale systems.

### Hydrogen Peroxide Detection Mechanism under Thin-Layer Conditions

The catalytic behavior of PB in the H_2_O_2_ reduction is primarily governed by its redox state and, despite the extensive literature on the catalytic mechanism of the PB adduct,^19^ elucidating its redox dynamics under specific experimental conditions remains crucial for the present study. According to the following reactions, when maintained in their oxidized forms (PB and BG), the material is catalytically inactive in the H_2_O_2_ reduction. Conversely, upon electrochemical reduction (eq 1), PW becomes capable of donating electrons to H_2_O_2_, thereby initiating its spontaneous catalytic reduction (eq 2). This reaction converts PW into PB, which is subsequently regenerated to PW through the electrochemical reduction driven by the negative polarization of the electrode.^37^ The resulting redox cycle between PB and PW produces a faradaic current directly proportional to the H_2_O_2_ concentration.

1 $${PB}_{(s)}+K_{(aq)}^{+}+e^{-}\to {PW}_{(s)}$$

2 $${PW}_{(s)}+H_{2}O_{2}\to {PB}_{(s)}+2{OH}^{-}$$

To ensure that the recorded electrochemical response originated solely from the interior of the CNP, rather than from any contribution of the external nanoring at the tip, two CVs were acquired with and without electrolyte solution inside the nanopipette (see Figure S3). Under these conditions, no faradic response was observed, and only background noise was detected. This result indicated that the external ring is not effectively coated with PB-NiHCF, confirming that the electrochemical signal arises from the inner walls of the modified CNP. Next, the performance of the PB-Ni CNP toward H_2_O_2_ reduction was systematically examined by CV and chronoamperometry at different experimental conditions.

Figure 3a shows three consecutive CV scans acquired immediately after electrode immersion (*t* = 0 s) in a 1 mM H_2_O_2_ solution (pH 4.3) within the potential window from +0.45 V to −0.20 V at 50 mV s^–1^. The PB-Ni CNP, serving as the WE, was prefilled with supporting electrolyte by capillary action and subsequently immersed in the H_2_O_2_ solution to establish the electrochemical cell and initiate the measurements. More positive potentials were deliberately excluded from the experiment, as the NiHCF layer promotes catalytic oxidation of H_2_O_2_,^41^ which is beyond the scope of the present study. No significant variation was observed between the scans, suggesting that no redox reaction related to H_2_O_2_ reduction occurs. Importantly, the interpretation of the results requires evaluation of both the geometrical and chemical nature of the CNP. Considering conventional macroelectrodes as a reference model, electroactive species are continuously replenished from the bulk solution to the electrode surface by diffusion, without any physical constraint. In the case of CNPs, the WE corresponds to the carbon-coated inner walls of the nanopipette tip, with diameters of only a few tens of nanometers and capable of confining extremely small solution volumes, typically from femtoliters to picoliters. Although the aliquot of sample (containing or not the analyte) inside the nanopipette remains in contact with the bulk solution through the open tip during the electrochemical measurement, the nanometric dimensions of the opening affect the supply of electroactive species from the bulk to the inner part of the CNP. Accordingly, mass transport from the bulk solution to the inner CNP tip must be considered, as it could become significantly delayed by the geometry of the nanopipette.

**Figure 3:**
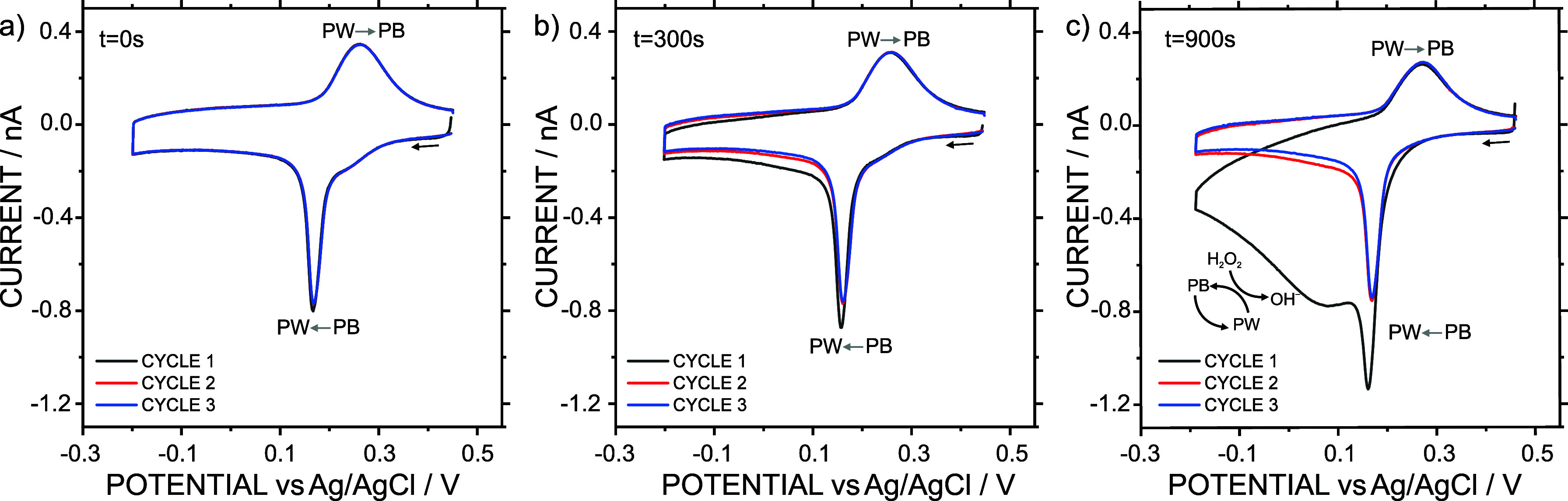
CVs of PB-Ni CNPs exposed to 1 mM H_2_O_2_ solution within the voltage range from +0.45 V to −0.2 V at a scan rate of 50 mV s^–1^, at different waiting times at 0.3 V: a) *t* = 0 s, b) *t* = 300 s, c) *t* = 900 s. Initial potential: + 0.45 V, forward scan from +0.45 V to −0.2 V, turning potential −0.2 V, backward scan from −0.2 V to +0.45, end potential +0.45 V.

To demonstrate this, a set of CVs in the presence of H_2_O_2_ was recorded after different waiting (or accumulation) times (*t* = 0, 300, 900 s) to allow H_2_O_2_ to diffuse into the tip. For 300 s, slight variations appeared in the cathodic region (Figure 3b), being consistent with the catalytic reduction of H_2_O_2_ by the PB polycrystal in its PW-reduced state.^42,43^ Note that the potential was held at 0.3 V, well above its open-circuit potential (∼0 V), to ensure that the coated material remained in its PB state and preventing spontaneous reduction of H_2_O_2_ during the accumulation of the analyte inside the CNP tip.^44^ After 900 s, a drastic current increase in the PW region was observed (Figure 3c), suggesting that more H_2_O_2_ is reduced on the WE and reinforcing the hypothesis that H_2_O_2_ requires time to diffuse from the bulk solution to the nanopipette tip. Importantly, after the first two CV cycles, the current returns to the background level, which likely means that all the electroactive species confined within the CNP tip undergo complete catalytic reduction (Figure 3c). This behavior is characteristic of nanoconfined redox-active compounds in CNPs, where the response is under a thin-layer regime.^13,45^

To further confirm this behavior, we extended the study to nine different waiting times (*t* = 0, 30, 60, 120, 300, 360, 480, 600, 900 s) in the absence and presence of bulk H_2_O_2_ (4 mM concentration). Then, CVs were recorded in the potential window from +0.45 V to −0.2 V at a scan rate of 50 mV s^–1^, by maintaining the WE potential at 0.3 V during the accumulation of the analyte before each measurement. The difference between the peak currents at 0.13 V (corresponding to PW) of the first and third cycles (Δ*i*) versus the waiting time was selected as the criterion to evaluate the time-dependent response evolution. Accordingly, Figure 4a shows the dependence of the waiting time on the defined analytical response Δ*i*. In the presence of H_2_O_2_, Δ*i* progressively increases with the time, indicating that a finite delay is required before a measurable analytical response is observed. In contrast, in the absence of H_2_O_2_, the signal remains nearly constant over the entire time window. This comparison suggests that the observed response arises from the diffusion-driven accumulation of H_2_O_2_ within the nanopipette, which requires sufficient time to reach the active region at the CNP tip. Additional experiments were conducted to elucidate such a time-dependent response, where the Δ*i* was plotted as a function of the H_2_O_2_ concentration, at different waiting times (Figure S4). Accordingly, Figure S5a shows the evolution of Δ*i* with the H_2_O_2_ concentration at different waiting times. Plotting the slope of each linear fit versus the waiting time (Figure S5b) revealed a clear relationship between the waiting time, influencing H_2_O_2_ diffusion (and thus its accumulation) into the nanopipette, and the analytical response. Further details are available in Section 3 of the Supporting Information.

**Figure 4:**
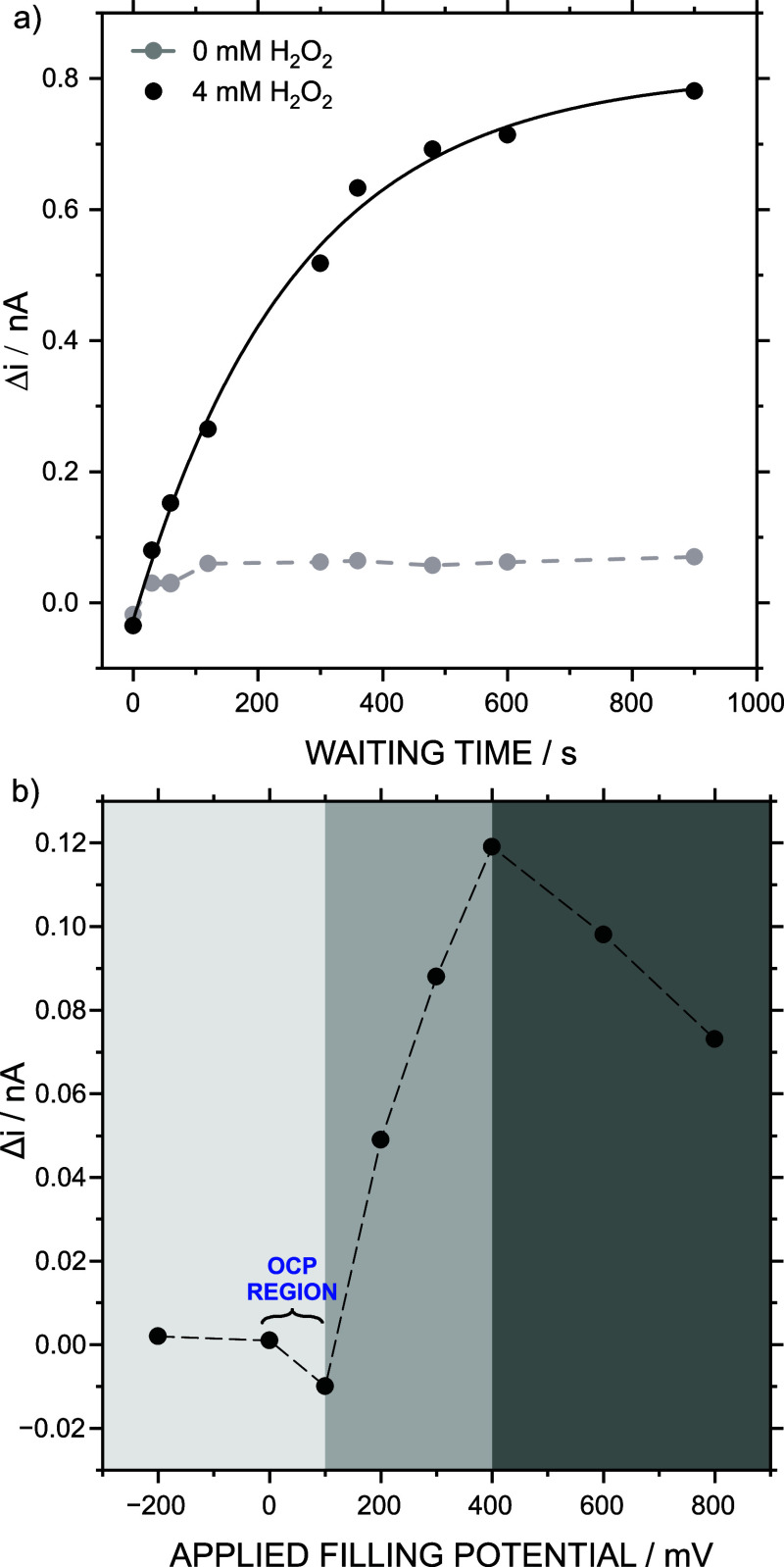
a) Variation of the analytical response (Δ*i* at 0.13 V) as a function of the waiting time for a PB-Ni CNP in the absence (gray dots) and presence of H_2_O_2_ (4 mM concentration) (black dots). b) Plot of the Δ*i* value as a function of the applied filling potential obtained in 500 μM H_2_O_2_.

Subsequently, experiments were carried out by controlling the volume of liquid (sample) inside the nanopipette using a pressure controller to apply positive or negative pressure at the tip level. Applying negative pressure to the empty pipet allowed for reaching a defined volume from the bulk solution directly into the CNP tip, thereby avoiding the waiting time normally required for the diffusion of the electroactive species from the bulk into the confined volume. Under this condition, the potential applied during the filling step becomes a key parameter, as it defines the redox state of PB. It may trigger the catalytic reduction of H_2_O_2_ before the central measurement, compromising the analytical output and the reliability of concentration determination in a quantitative step. Consequently, the potential imposed on the WE during nanopipette filling from the bulk solution was systematically investigated by CV.

Eight filling potentials were selected within the range from −200 mV to +800 mV vs Ag/AgCl to fill the nanopipette with 500 μM H_2_O_2_ solution, aiming at exploring all the redox forms of the PB structure. The observed CVs are reported in Figure S6. As a metric of the filling-potential effect, the Δ*i* was plotted against the applied filling potential. The results are depicted in Figure 4b. Three main regions can be discriminated. The first region, between −200 mV and +100 mV, shows Δ*i* values close to 0 nA, suggesting that the spontaneous reduction of H_2_O_2_ occurs during the sample loading, and thus avoiding its detection during the posterior CV experiment. The film is maintained (at least partially) in the PW state, triggering the early reduction of the H_2_O_2_ during the nanopipette loading before the CV measurement. Interestingly, this potential range includes the OCP at which the PB-Ni CNP normally runs. In a second region, from 100 mV to 400 mV, the curve shows an increase until reaching a maximum Δ*i* value at +400 mV, indicating that the film was gradually shifted from the reduced PW state to the oxidized PB state. Therefore, less H_2_O_2_ was catalytically consumed inside the tip before the voltammetric measurement, resulting in a higher Δ*i* value. In other words, the closer the filling potential is to the PB redox region, the more the catalyst remains in its oxidized state during the filling of the CNP with the analyte, preserving the analyte for subsequent detection. In the third region, from 400 mV to 800 mV, a diminishing of Δ*i* was observed, which is likely attributed to the catalytic oxidation of the H_2_O_2_ promoted by NiHCF structures, as documented in the literature.^41^

Additional studies about the influence of the applied filling potential on the detection performance were carried out by DPSC. In each experiment, a defined voltage was applied during the filling of the CNP, after which the DPSC routine was initiated. DPSC consisted of a +0.40 V voltage step for 10 s to maintain the electroactive H_2_O_2_ in its oxidized form after nanopipette filling, followed by a 200 s step at −0.20 V to promote H_2_O_2_ reduction. To assess possible background contributions at each applied filling potential DPSC experiments were recorded in the presence and absence of H_2_O_2_ (Figure 5). Figure 5a shows the chronoamperometric responses obtained at different filling potentials in the absence of H_2_O_2_ (i.e., buffered solution). A cathodic current spike was observed in the DPSC response when the potential was switched from +0.4 V to −0.2 V, followed by a steady-state current that reached the same value for all the investigated potentials. In addition, the area under the peak accounts for the charge value for both the capacitive component and the faradaic contribution arising from the PB → PW redox transition. Thus, no significant variations were observed upon changing the filling potential, indicating that the effect of this parameter in the background signal and PB redox switching was negligible. In contrast, Figure 5b presents a markedly different behavior in the presence of H_2_O_2_ (500 μM), where the amplitude of the reduction peak depended on the applied filling potential.

**Figure 5:**
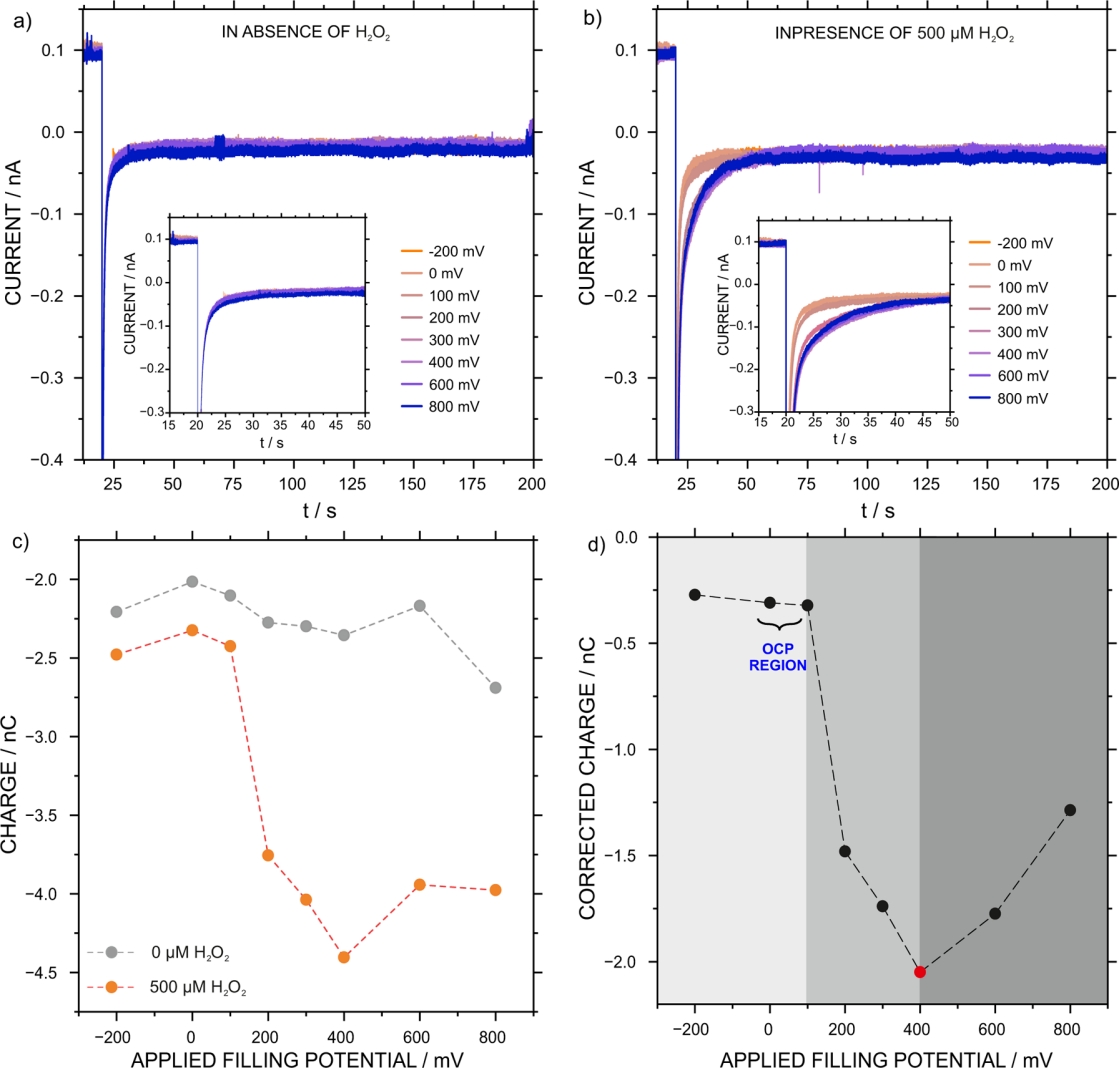
Double-potential step chronoamperometry at different applied filling potentials. a) Response in the absence of H_2_O_2_. b) Response in the presence of 500 μM H_2_O_2_. c) Plot of the charge obtained from DPSC measurement at different applied filling potentials in the absence (gray line) and presence (orange line) of 500 μM H_2_O_2_. d) Plot of the corresponding corrected charges. The three potential windows are indicated with gradually increasing gray color.

To provide further insight into this phenomenon, the cumulated charge (*Q*) associated with each DPSC signal was determined by integrating the chronoamperograms. Figure 5c plots the *Q* values obtained in the absence (gray dots) and presence (orange dots) of 500 μM of H_2_O_2_ as a function of the applied filling potential. Despite slight fluctuations in the charge values arising from the background, a significant trend characterized by a net charge drop was observed in the presence of H_2_O_2_, due to its reduction within a specific filling potential interval. To eliminate the capacitive and intrinsic faradic contribution due to the PB → PW transition, the charge values from the blank experiments were subtracted from the corresponding *Q* values in the presence of H_2_O_2_. The resulting net faradaic charge associated with the reduction of H_2_O_2_ was then plotted versus the applied filling potential (Figure 5d). In total accordance with the CV response reported in Figure 4b, the pattern found for the corrected charge versus the applied filling potential depicts three main potential regions.

When the nanopipette was filled under potentials within the H_2_O_2_ reduction window (between −200 mV and +100 mV), the analyte was expected to be fully electrolyzed prior to the reduction step and therefore, no difference in *Q* should be observed between the blank and the H_2_O_2_ sample. In practice, after subtracting the two DPSC curves, only a small residual charge (∼0.3 nC) was obtained, which we attribute to minor nonfaradaic contributions and/or fitting/integration uncertainty rather than to incomplete pre-electrolysis of H_2_O_2_. Then, the potential range between +100 mV and +400 mV is characterized by a net decrease in the charge due to the reduction of the H_2_O_2_, in agreement with the PB redox state. At more positive potentials, the increase in the charge suggests that less H_2_O_2_ is reduced, in accordance with the catalytic performance of the embedded NiHCF toward the oxidation of the H_2_O_2_. Finally, the effect of the reduction potential step was also investigated by performing DPSC measurements in the presence of 500 μM H_2_O_2_ at five different reduction potentials (−300, −200, −100, −50, and 0 mV): as shown in Figure S7, comparable *Q* values were obtained in the −200 to −50 mV range. All in all, the DPSC outcomes and the consistent results obtained with CV clearly demonstrate that proper sampling of the analyte aliquot is a critical factor for reliable H_2_O_2_ sensing using PB-Ni CNPs systems.

### Investigation of the Analytical Performance of PB-Ni CNPs toward Hydrogen Peroxide

The sensing performance of the PB-Ni CNP toward H_2_O_2_ was evaluated by DPSC at two pH values, 4.3 and 7.4, maintaining the filling potential at +0.4 V. Thus, the potential waveform consisted of a 10 s preconditioning step at +0.40 V followed by a 150 s reduction step at −0.20 V, as optimized in the previous section. The charge associated with H_2_O_2_ reduction was obtained by integrating the cathodic peak, taking the median value of the steady-state current as the baseline reference. Then, the resulting charge values were plotted as a function of H_2_O_2_ concentration. Figure 6a displays the DPSC responses obtained at pH 4.3 at the different concentration levels. The amplitude of the cathodic peak increases proportionally with the concentration. Up to 500 μM, the steady-state current converges to approximately the same value, which is diagnostic of the thin-layer regime due to the depletion of the confined analyte inside the nanopipette. The steady-state current remained more negative only at 1000 μM, attributable to a partial diffusion-controlled behavior due to the relatively high bulk concentration of hydrogen peroxide. Further investigations on the thin-layer regime (Figure S8) carried out by DPSC measurements demonstrated that the reduction of 200 μM H_2_O_2_ initially follows a diffusion-controlled behavior, and subsequently reaches complete consumption of the confined analyte, leading to a charge plateau and confirming the predominance of thin-layer electrochemistry. Assuming exhaustive electrolysis of the confined H_2_O_2_ aliquot, the integrated faradaic charge can be used to estimate an apparent confined volume through Faraday’s law (*Q* = nFCV). The resulting apparent volume calculated from the calibration data set (Figure 6a,b) was 17.7 ± 1.3 pL (Supporting Information, Table S1), which is consistent with the expected picoliter range of carbon nanopipettes.^13,46^

**Figure 6:**
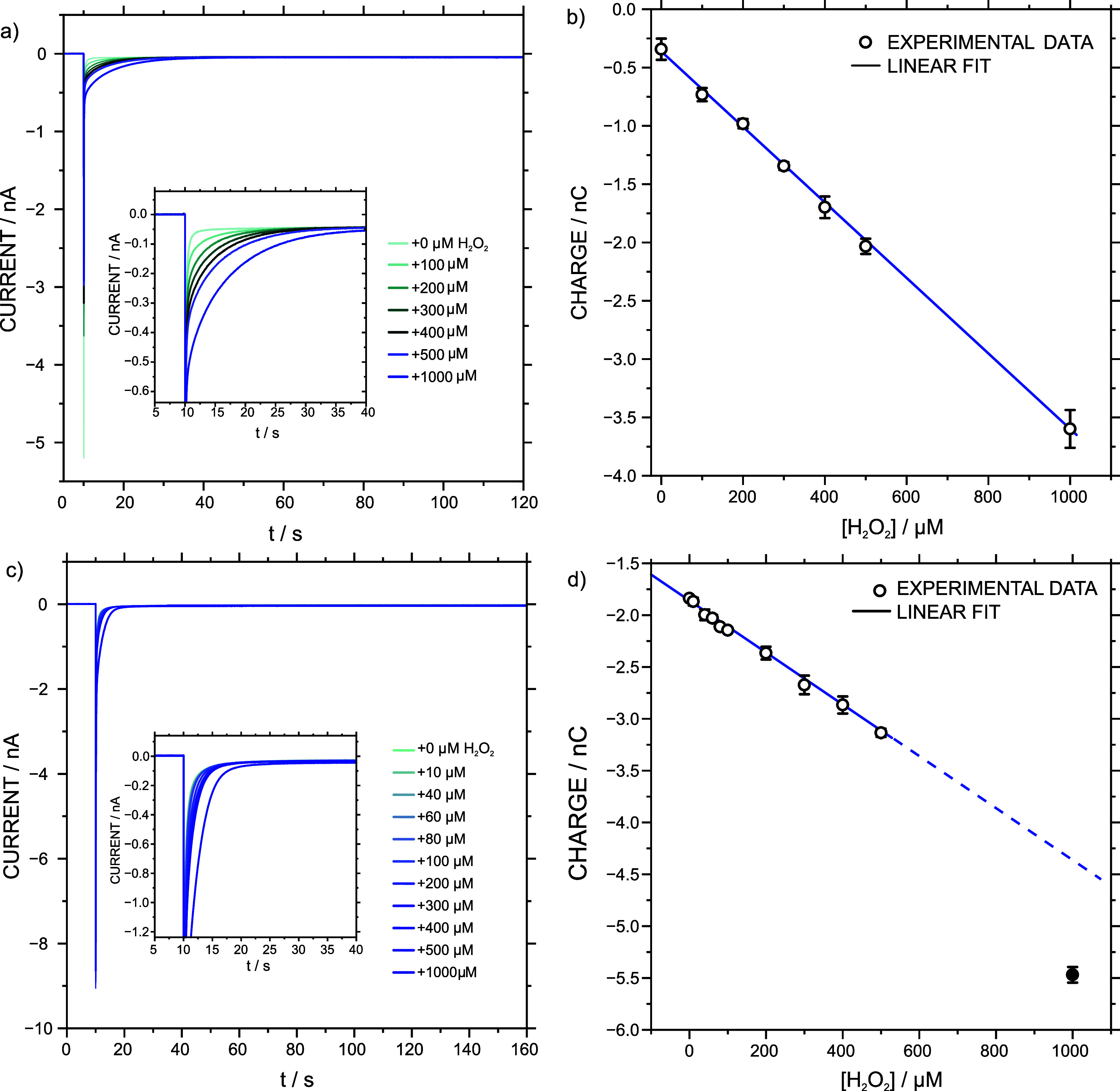
a) Double-potential step chronoamperometry responses related to the PB-Ni CNP tested at increasing H_2_O_2_ concentrations at pH 4.3. b) Corresponding calibration graph obtained from the charge values. c) Double-potential step chronoamperometry responses related to the PB-Ni CNP tested at increasing H_2_O_2_ concentrations at pH 7.4. d) Corresponding calibration plot obtained from the charge values. Error bars represent the standard deviation obtained from triplicate measurements (*n* = 3) at each concentration level.

Figure 6b depicts the calibration plot obtained from the *Q* values at increasing concentration. Error bars indicate the standard deviation from triplicate measurements (*n* = 3) at each concentration level. The data follow a linear regression (*R* ^2^ = 0.997) between 0 and 1000 μM according to the equation

$$Q=\left(-3.00\pm 0.08\right)10^{-3}c+\left(-0.356\pm 0.024\right)$$

where *Q* is the charge expressed in nC, and *c* is the H_2_O_2_ concentration expressed in μM. The bias from 0 nC in the intercept (−0.356 ± 0.024 nC) is connected to the capacitive and faradic contributions due to the PB → PW redox transition in the absence of the analyte. The limit of detection (LOD, 83.0 μM) and the limit of quantification (LOQ, 276.0 μM) values were calculated according to the 3 × *sb*/*m* and 10 × *sb*/*m* criteria, where *sb* corresponds to the standard deviation obtained with 7 different measurements in the absence of H_2_O_2_, and *m* represents the slope of the calibration plot. The DPSC response for 500 μM of H_2_O_2_ obtained from 5 different measurements with the same nanopipette performed on the same day provided an RSD_*n*=5_ = 2.93% (relative standard deviation), showing the acceptable reproducibility of the protocol used for the PB-Ni CNPs sensor and chronoamperometric transduction.

Also, the CNP was tested in PB buffer (KH_2_PO_4_/K_2_HPO_4_, KCl 0.3 M) at pH 7.4, allowing to assess the stability of the PB-NiHCF system at physiological pH. Figure 6c shows the DPSC profiles recorded in the 0–1000 μM H_2_O_2_ concentration range. In agreement with the calibration performed at pH 4.3, a constant increase in the cathodic peak magnitude proportional to the H_2_O_2_ concentration is observed, with the current plateau reached up to 500 μM. A pronounced negative drift of the steady-state current appears at 1000 μM, likely due to the excessive amount of H_2_O_2_ forcing diffusive transport of the analyte toward the inner tip of the CNP. Figure 6d presents the corresponding calibration plot, showing a linear trend (*R* ^2^ = 0.996) between 0 and 500 μM, described by the equation

$$Q=\left(-2.53\pm 0.05\right)\times 10^{-3}c+\left(-1.87\pm 0.013\right)$$

Notably, the two calibrations at different pH levels exhibited comparable sensitivity, as observed in the slope values of the corresponding calibration curves. However, at pH 7.4, the data point at 1000 μM does not align with the linear fit (and was therefore excluded from the regression), likely due to the excessive generation of OH^–^ anions produced during H_2_O_2_ reduction: this may induce irreversible damage to the thin electroactive PB-NiHCF film. This behavior is demonstrated in the CVs reported in Figure S9, where a partial decrease in the PB domain is observed upon exposing the PB-Ni CNP from pH 4.3 to pH 7.4, indicating a partial degradation of the film under neutral conditions. LOD and LOQ values were calculated as previously described, resulting in 28.9 and 85.9 μM, respectively. Finally, the calibration points showed excellent repeatability, as evidenced by a low RSD_*n*=5_ of 1.26%, confirming the good precision of the method at pH 7.4.

The reversibility of the PB-Ni CNP toward H_2_O_2_ sensing was evaluated by cycling the response six times between 0 and 500 μM of H_2_O_2_ concentration at pH 4.3. Figure S10a shows the charge coming from each measurement versus the number of cycles performed by the sensor, evidencing an acceptable alternation between the two concentration levels, with an average charge of 0.59 ± 0.11 nC at 0 μM and 1.54 ± 0.15 nC at 500 μM, with consistent recovery of the signal at each cycle and no significant loss of amplitude. The slight positive drift observed in the data with the cycles is likely attributable to the partial cleavage of the PB domain inside the nanopipette due to the catalytic reduction of the hydrogen peroxide.^33,43^ This finding is supported by the CVs carried out before and after the procedure in Figure S10b, where a slight current diminution in the PB voltage range domain is evidenced.

The selectivity was investigated at pH 4.3 and 7.4 by comparing the amperometric response obtained for 500 μM of H_2_O_2_ in the presence of potential interferents such as d-(+)-Glucose (Gluc), Uric Acid (UA), and l-Ascorbic Acid (AA), which are commonly found in biological samples. At pH 4.3 (Figure S11a), these compounds did not cause relevant signal variation within the *x̅* ± 3SD and *x̅* ± 1.5SD threshold limits, demonstrating that the method exhibits acceptable selectivity. At pH 7.4 (Figure S11b), similar results were obtained, further confirming the suitability of the CNP usage under physiological pH. Although not directly demonstrated, the loading step at +0.40 V of the DPSC may act as an electrochemical pretreatment of the incoming solution, oxidizing electroactive interferents (such as ascorbic and uric acids) before the H_2_O_2_ reduction stage.

An additional calibration experiment performed on a third independent PB-NiHCF-modified CNP is reported in the Supporting Information (Figure S12), providing a preliminary assessment of the reproducibility of the proposed methodology. Minor variations in the integrated charge values are expected due to differences in the confined sample volume and the fabrication of independently prepared CNPs.

## Conclusions

This study establishes the integration of Prussian Blue (PB) modified with nickel hexacyanoferrate (NiHCF) inside carbon nanopipettes (CNPs) operating under thin-layer electrochemical conditions. The NiHCF overlayer stabilizes the PB film response toward H_2_O_2_ under physiological conditions, mitigating the loss of electroactivity typically observed for PB in neutral media and enabling reliable operation at pH 7.4. Considering the intrinsic catalytic properties of both electroactive materials, H_2_O_2_ sampling conditions were thoroughly examined, proving to be a critical factor for the electrochemical detection. Loading procedures performed at open-circuit potential or filling potentials below 0.3 V led to the premature electrolysis of H_2_O_2_ due to the presence of Prussian White (the reduced form of PB), while more positive loading potentials preserved the analyte for subsequent readout. Considering this, the optimization of a protocol based on double-potential step chronoamperometry (DPSC) enabled precise control over this aspect, ensuring H_2_O_2_ delivery into the nanometric inner tip and its subsequent exhaustive consumption within time scales of tens of seconds. Specifically, the sensor exhibited a limit of detection of 28.9 μM, acceptable repeatability (RSD_*n*=5_ = 1.26%), and negligible signal variation in the presence of common biological interferents. The linear response toward H_2_O_2_ at physiological pH in the range from 28.9 μM to 500 μM makes it an excellent candidate for single-cell studies. Beyond the possibility of H_2_O_2_ quantification, this work provides a general approach for operating CNPs under thin-layer electrochemical conditions, highlighting the role and implications of nanoconfined environments for analytes of biological interest. This methodological framework strengthens the applicability in real biological samples and opens the way for future research toward real-time monitoring in living cells as well as the development of coulometric nanosensors for reactive oxygen species (ROS). Beyond the methodological framework established herein, future investigations involving single-cell measurements in complex biological environments are required to further assess the performance and applicability of the PB-NiHCF CNP platform, and to address challenges such as tissue penetration, measurement noise, and local analyte transport phenomena.

## Supplementary Material



## Notes

PB-Ni stability evaluation; electroactive surface investigation; time-dependent analysis; applied potential effect; thin-layer behavior investigation; pH effect; reversibility and selectivity (PDF)
