# Supplementary material for: Nanoelectroanalysis with Carbon Nanopipettes Based on Prussian Blue-NiHCF for Hydrogen Peroxide Sensing
Source: Anal Chem. 2026 Jun 25;98(30):22348–58. doi: 10.1021/acs.analchem.6c02551 (PMC13449225; doi:10.1021/acs.analchem.6c02551)
Supplement: Supplementary file 1 [file ac6c02551_si_001.pdf]

Supporting information for:

Nanoelectroanalysis with carbon nanopipettes based on Prussian Blue-NiHCF for hydrogen peroxide sensing

Antonino Biagio Carbonaro[1], Gregorio Laucirica[1], Gastón A. Crespo[1,2], Maria Cuartero[1,2,*]

[1] UCAM-SENS, Universidad Católica San Antonio de Murcia, UCAM HiTech, Avda. Andrés Hernández Ros, 1, 30107, Murcia, Spain.
[2] Department of Chemistry, KTH Royal Institute of Technology, Teknikringen 30, SE-114 28, Stockholm, Sweden.

*mariacb@kth.se

Table of contents

1. Stability evaluation of the electrodeposited films SI-3
2. Replicate PB-Ni CNP fabrication SI-4
3. Electroactive surface investigation SI-5
4. Time-dependent diffusion of hydrogen peroxide inside PB-Ni CNPs SI-5
5. Effect of the applied potential..SI-8
6. Reduction potential investigation on DPSC experiment SI-8
7. Thin-layer behaviour investigation..SI-9
8. pH influence on the PB-NiHCF CNPs system SI-10
9. Reversibility and selectivity...SI-11
10. References SI-12

1. Stability evaluation of the electrodeposited films

To evaluate the effect of NiHCF on the stability of the PB thin film electrodeposited inside the CNP tip, repetitive CV cycles were performed in PB buffer (0.05 M, pH 7.4), both in the presence and in the absence of NiHCF. **Figure S1a** shows the electrochemical behavior of the PB–CNP system after the stabilization procedure described in the main text. In the first scan (black line), the typical PB redox peaks are observed. However, from the second cycle (red line), a new redox plateau appears around 0.2 V, accompanied by a rapid decrease in the PW and BG signatures at approximately 0.13 V and 0.75 V, respectively. This trend becomes more evident in successive cycles, where the PW and BW peaks progressively vanish and are replaced by a pair of symmetric oxidation and reduction peaks at 0.2 V. Considering similarities with previously reported voltammetric profiles [1], this symmetric pair of peaks could be associated with either the stabilization of a different form of Prussian Blue or related PB analogues. However, considering the progressive disappearance of the Berlin Green (BG) redox signature around +0.8V during repeated CV cycling, together with the limited stability of PB under physiological conditions arising from the strong interaction between hydroxyl ions and ferric sites, the progressive hydrolysis of the polycrystalline framework cannot be excluded[2]. On the other hand, the presence of the NiHCF structure provides enhanced stability, as evidenced by the consistent and reproducible voltammetric sweeps shown in **Figure S1b**, opening the way for future applications in physiological environments.

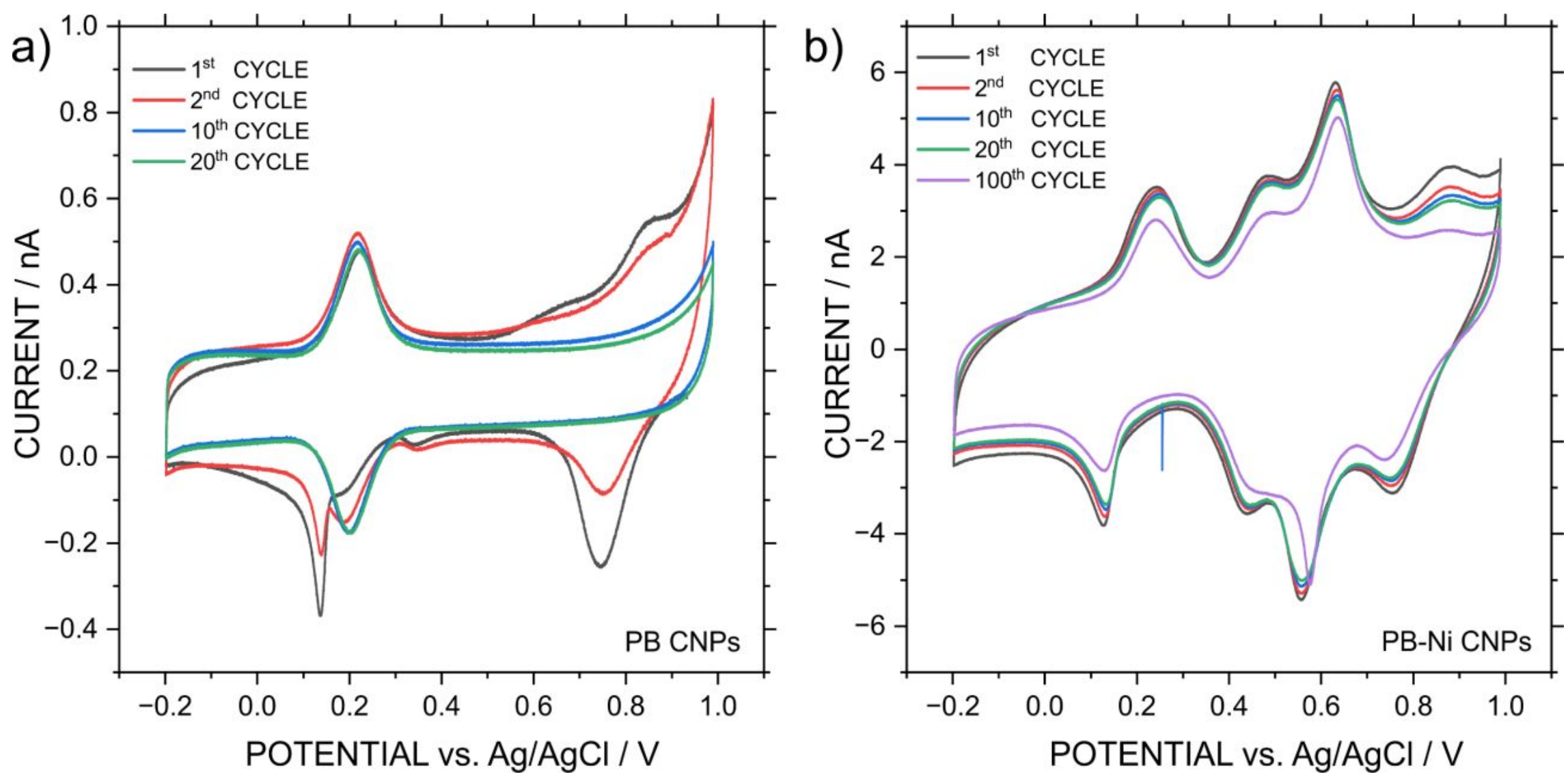

Figure S1: Stability experiment comparison performed by CV sweeping in the potential window +1.0/-0.2V at a scan rate of 100mV/s, for a) PB-CNP and b) PB-Ni CNP. The CVs were performed in 0.05M phosphate buffer pH 7.4

2. Replicate PB-Ni CNP fabrication

Figure S2 shows the results with two additional CNPs, labelled as CNP2 and CNP3, whereas the experiment discussed in the main manuscript corresponds to CNP1 (**Figure 2**). The main differences

observed between the datasets regards the current intensities recorded during the electrodeposition and stabilization steps. This variability could be associated to slight differences in the amount of precursor confined inside the CNP nanotip.

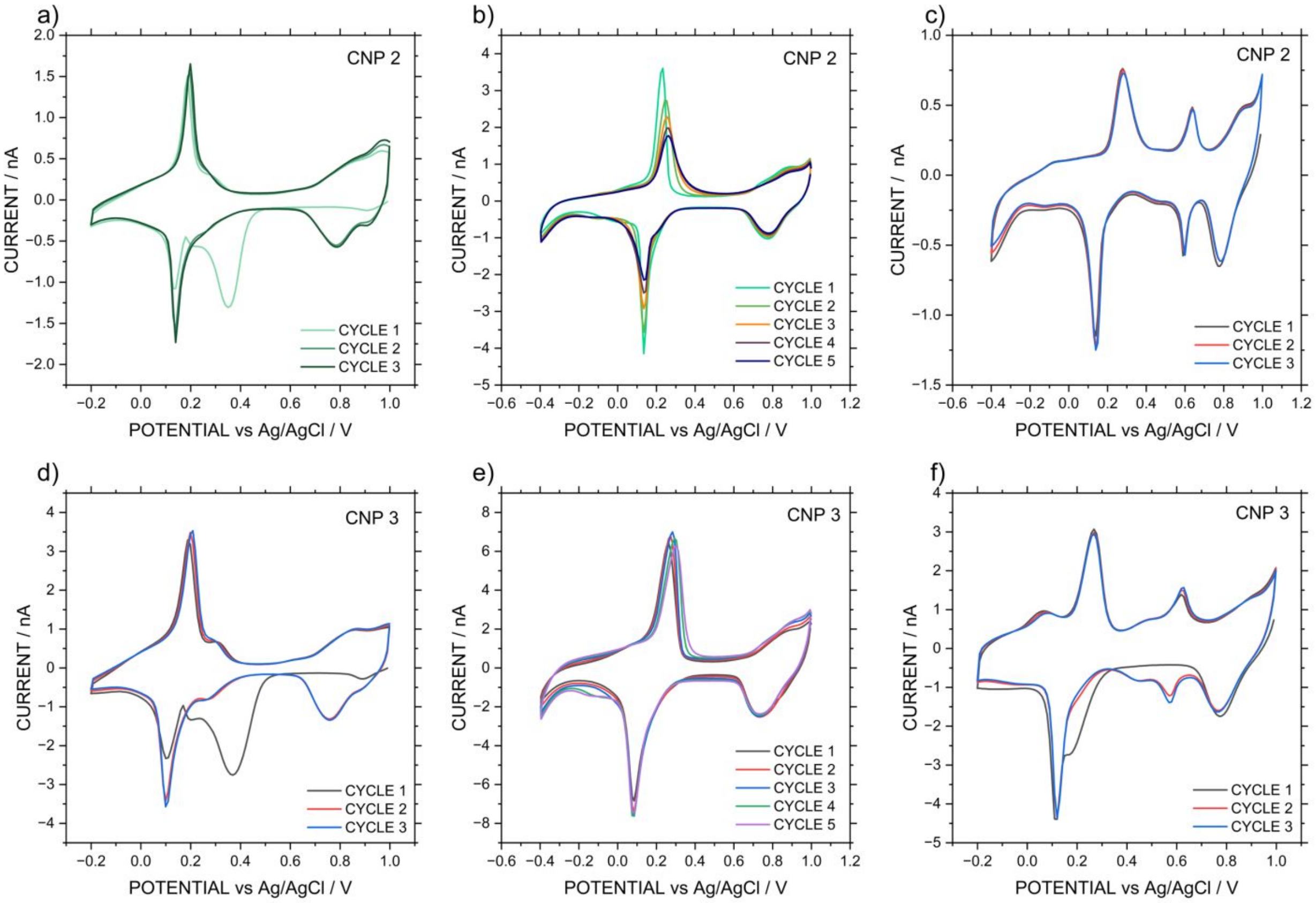

Figure S2: **a)** CVs observed for CNP 2 in the PB electrodeposition in CNPs tip performed by three CV scans at a scan rate of 50 mV s^{-1}. Initial potential: +1.0V, forward scan from +1.0V to –0.2V, turning potential –0.2V, backward scan from –0.2V to +1V, end potential 1.0 V. **b)** CVs observed for CNP 2 in the stabilization of the PB-CNPs**,** scan rate of 100 mV s^{-1}. Initial potential: +1.0V, forward scan from +1.0V to –0.4V, turning potential –0.4V, backward scan from –0.4V to +1V, end potential 1.0 V. **c)** CVs observed for CNP 2 in the Nickel hexacyanoferrate (NiHCF) electrodeposition in PB-CNPs tip performed by three CV scans at a scan rate of 50 mV s^{-1}. Initial potential: +1.0V, forward scan from +1.0V to –0.2V, turning potential –0.2V, backward scan from –0.2V to +1V, end potential 1.0 V. Same protocol concerning the **d)** PB electrodeposition, **e)** stabilization, and **f)** Nickel hexacyanoferrate (NiHCF) electrodeposition for CNP 3.

3. Electroactive surface investigation

To demonstrate that the electroactive surface of the modified PB–Ni CNPs originates solely from the inner tip surface, with a negligible contribution from the external tip ring, two CVs were recorded in the background electrolyte by comparing the empty and the filled nanopipette. As shown in **Figure S3a**, the empty configuration produces only background noise, indicating that the ring contribution can be considered negligible compared with that of the inner surface walls. The magnification shown in **Figure S3b** allows for clearer visualization, thereby supporting the contribution of the inner CNP part.

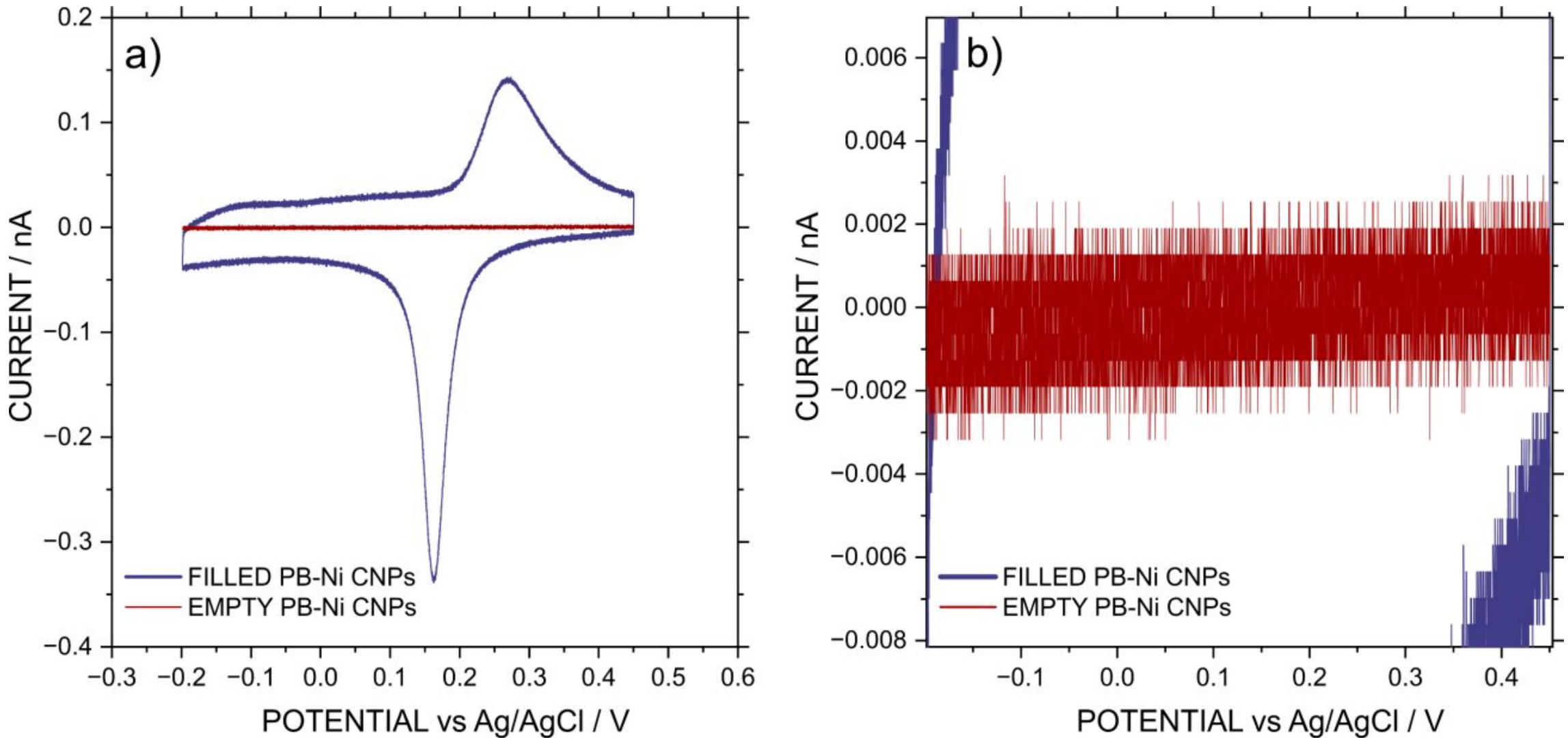

Figure S3: a) Cyclic voltammograms of PB-Ni CNPs recorded in the background electrolyte within the voltage range +0.45V/-0.2V at a scan rate of 50 mV s^{-1} of the filled (violet) and empty (red) nanopipette. **b)** magnification of the current signal produced by the empty nanopipette

4. Time-dependent diffusion of hydrogen peroxide inside PB-Ni CNPs

In order to further investigate the time-dependent diffusion of the analyte, we tested six different waiting time intervals (60–900 s) under bulk hydrogen peroxide concentrations (0–4 mM), while maintaining the potential at 0.3 V before the CVs. Then, cyclic voltammograms were recorded in the voltage range of +0.45 V/–0.2 V at a scan rate of 50 mV s^{-1}. For this purpose, the difference between the PW peak currents at 0.13 V of the first and third cycles (Δi, pA) versus H_2O_2 concentration (mM) was selected as the criterion to evaluate the time-dependent response (**Figure S4**). For each time value, an independent calibration curve was constructed and its slope, defined as the calibration sensitivity, was extracted for qualitative comparison purposes. **Figure S5a** shows that, as the waiting time increases, the analytical response as a function of concentration exhibits a progressively increasing trend. While the datasets recorded at shorter times (60 and 120 s) display a clear linear behaviour, slight deviations from linearity become evident at longer waiting times. For this reason, the linear trends at extended times are presented as qualitative guides to highlight the overall evolution of the response. The resulting slopes were then plotted as a function of the waiting time to illustrate

the time-dependent analytical response (**Figure S5b**).

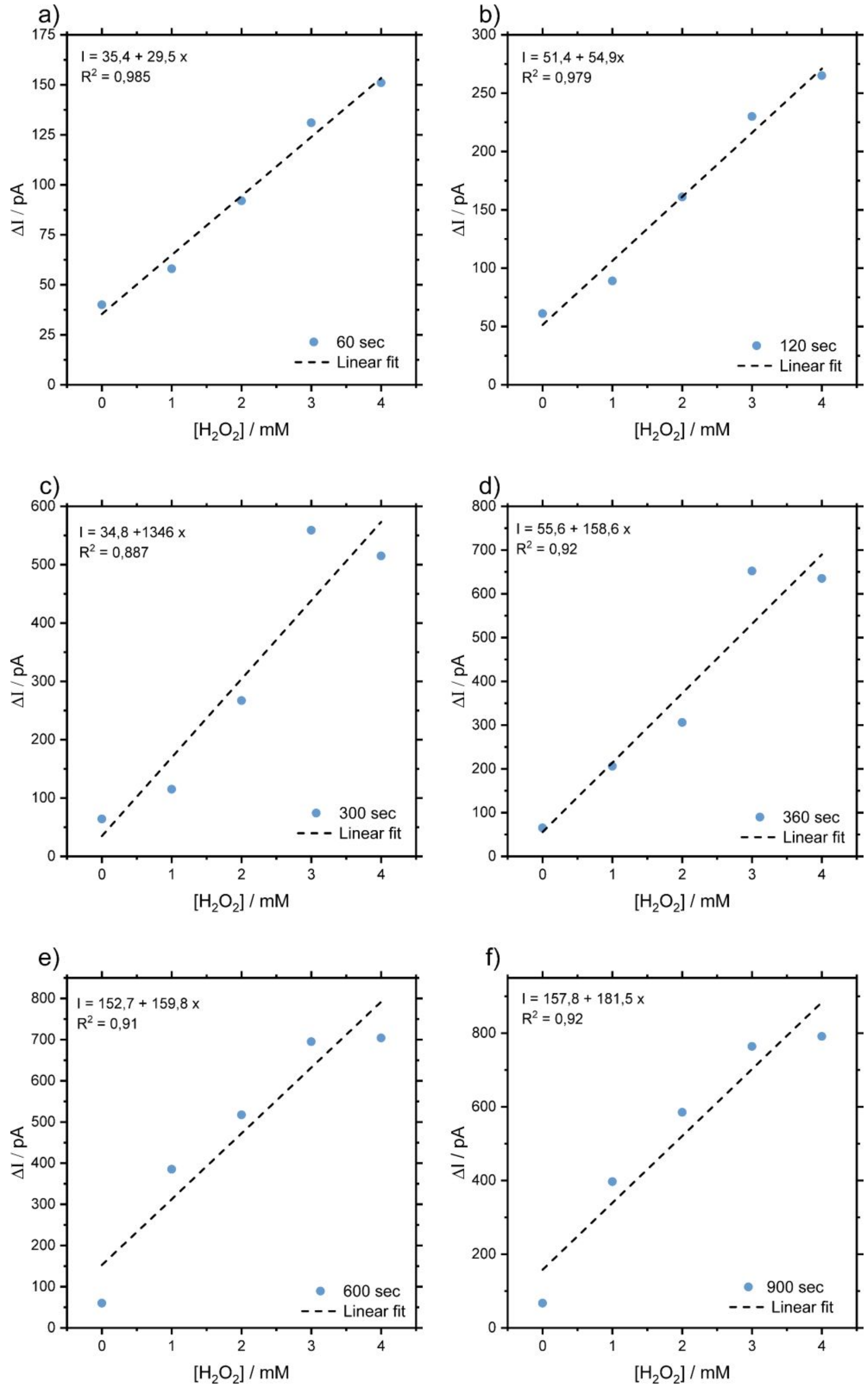

Figure S4: Difference in PW peak current (ΔI) between the first and third CV scans as a function of hydrogen peroxide concentration at increasing time. **a)** 60 s; **b)** 120 s, **c)** 300 s; **d)** 360 s; **e)** 600 s; **f)** 900 s.

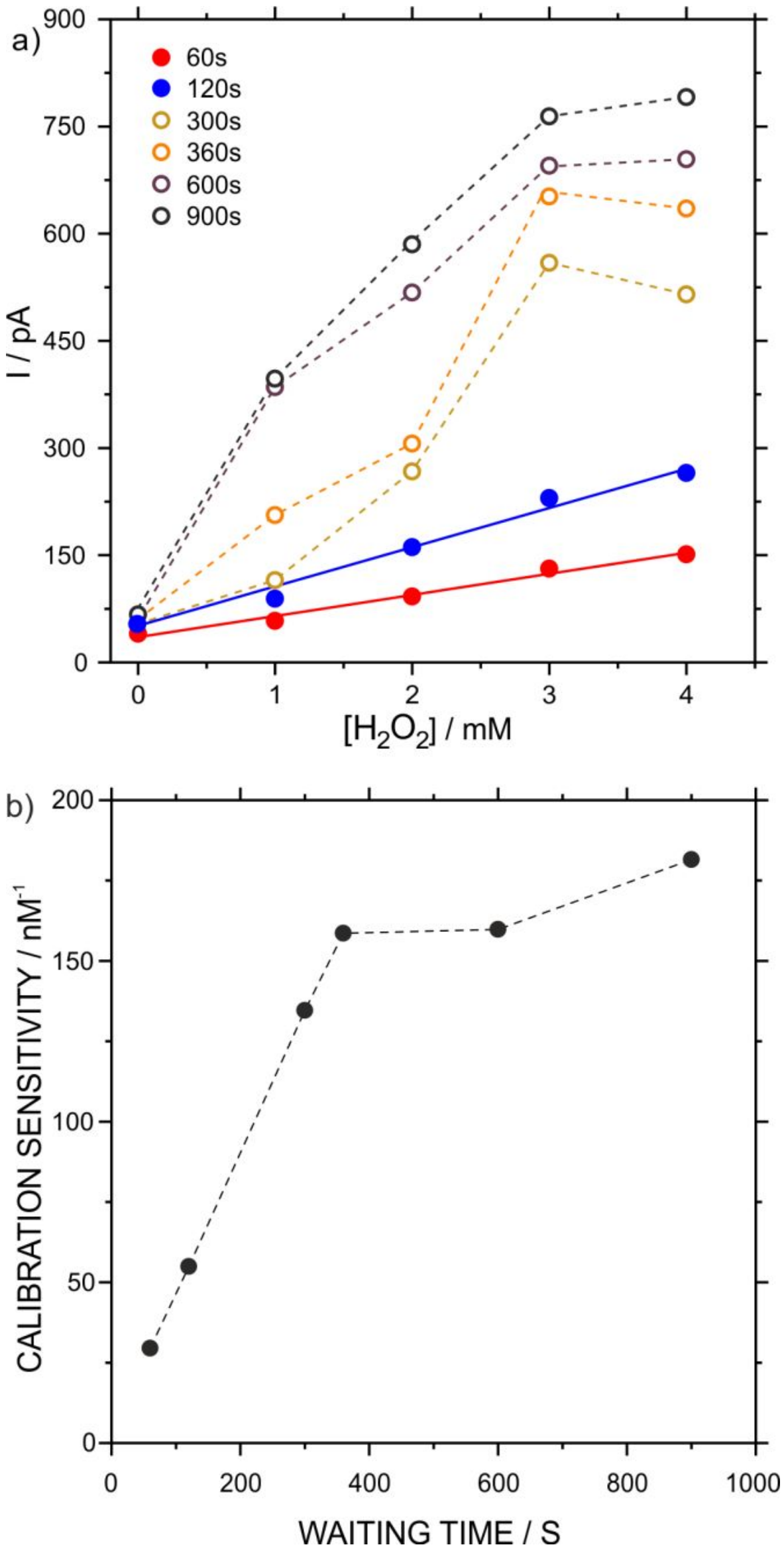

Figure S5: **a)** Cumulative calibration trends obtained at increasing waiting time vs. hydrogen peroxide concentration; **b)** behaviour of the calibration sensitivity as a function of the waiting.

5. Effect of the applied potential

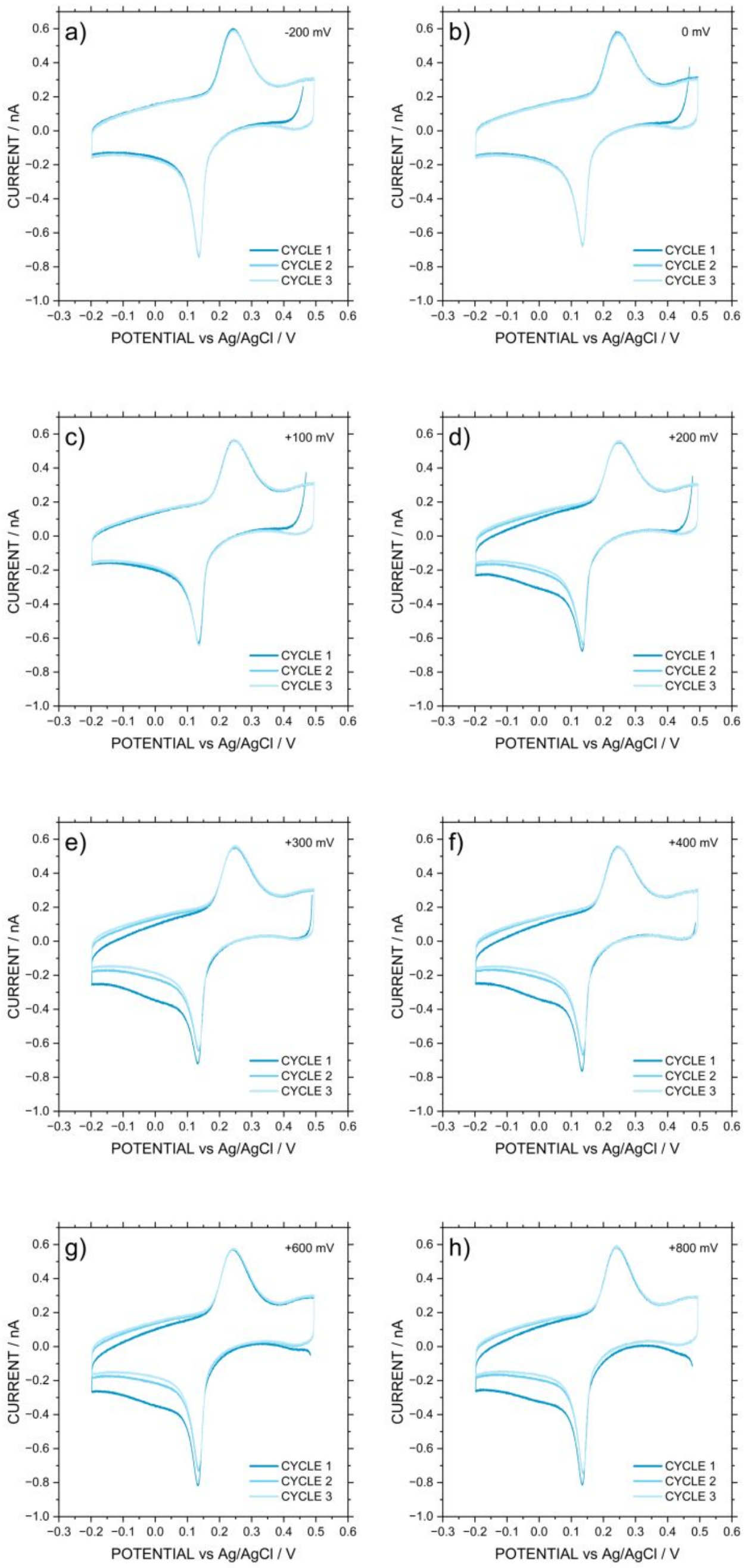

Figure S6: Cyclic voltammetry measurements recorded in 500 µM H_2O_2 by controlling the filling potential, expressed in mV, on the PB-Ni CNP system in the range +0,5V/-0.2V at the scan rate of 50 mV/s.

6. Reduction potential investigation on DPSC experiment

To evaluate the effect of the applied potential in the second step of the DPSC experiment on the reduction of 500 µM H_2O_2, pH=4.3, five different potentials (–300, –200, –100, –50, and 0 mV) were investigated, while maintaining the CNP loading potential at +400 mV. The integrated charge obtained in the presence of the analyte was corrected by subtracting the corresponding charge values recorded from blank measurements at the same potentials. As shown in Figure S6, except for –300 mV, all potentials display comparable corrected charge values, suggesting that the performance of

the PB–Ni CNPs remains essentially unchanged when PB is maintained in its reduced PW form. Interestingly, at –300 mV, the corrected charge is noticeably lower than at less negative potentials; however, the origin of this behaviour is not yet fully understood and is currently under investigation.

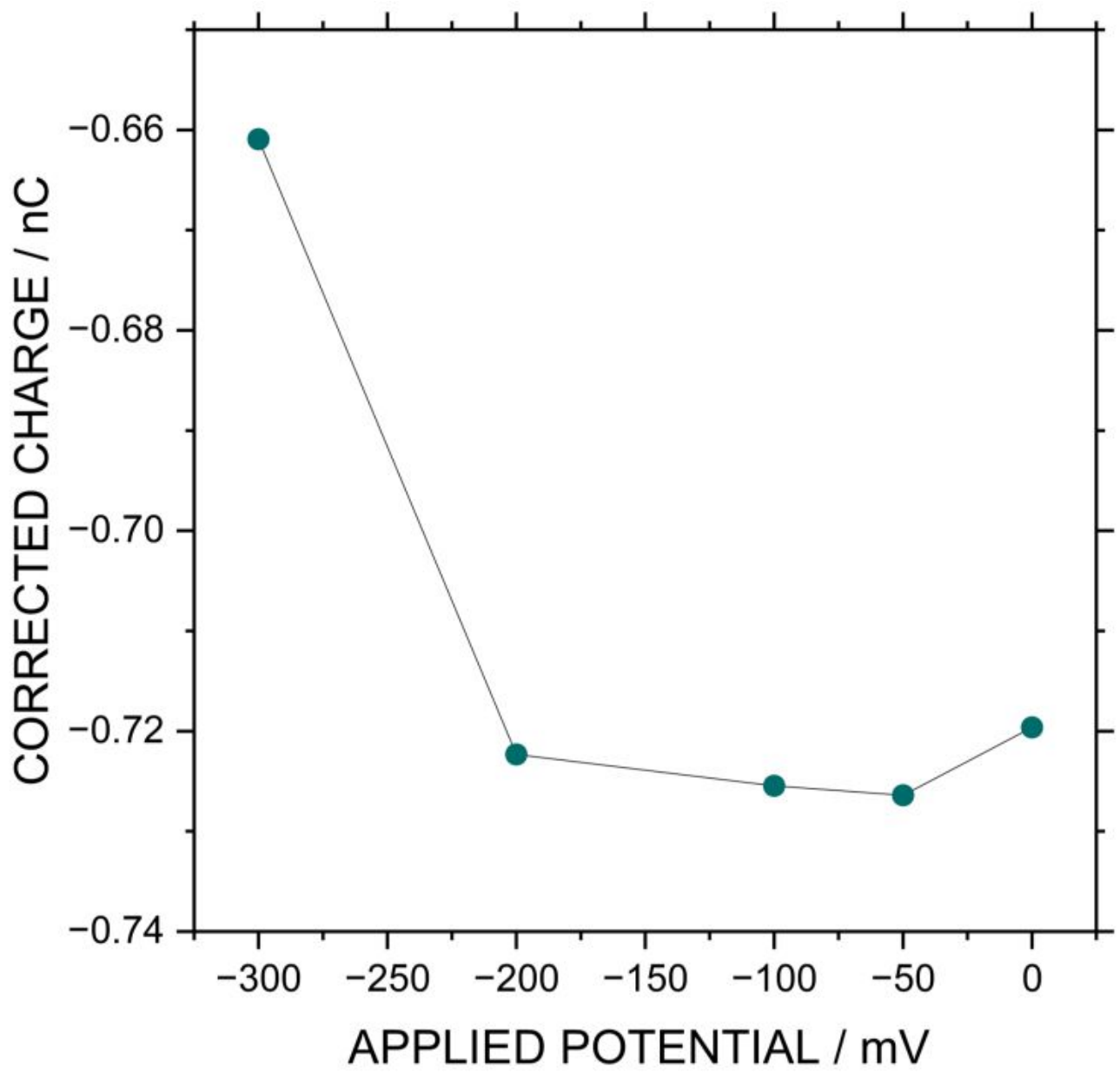

Figure S7: Corrected charge values (Q, nC) integrated from DPSC experiments performed at five different reduction potentials for hydrogen peroxide 500 µM, pH 4,3

7. Thin-layer behaviour investigation

Figure S7a reports the net faradic current associated with the reduction of the 200 µM H_2O_2 after the subtraction of the capacitive and the faradic contribution recorded for the blank. The chronoamperogram shows a rapid decay that approaches a steady state current of ~0 nA, with only a residual noise due to the experimental setup. **Figure S7b** shows the integrated corrected charge that reaches an apparent plateau from ~150s, suggesting that the total amount of the hydrogen peroxide confined within the CNP tip goes under reduction. In the Cottrell representation (**Figure S7c**), it is possible to note that at short times (right part of the graph) the experimental curve can be locally approximated by a Cottrell-type trend, dominated by semi-infinite diffusion (light blue dashed line). However, the trend strongly deviated from the Cottrellian behaviour at longer times, converging to the ideal limit of $i = 0$ (red dashed line, $y = 0$), once the confined hydrogen peroxide is totally

consumed. Thus, the overall analysis performed demonstrated that the PB-Ni CNP under investigation for the hydrogen peroxide detection is mostly governed by the thin-layer regime[3].

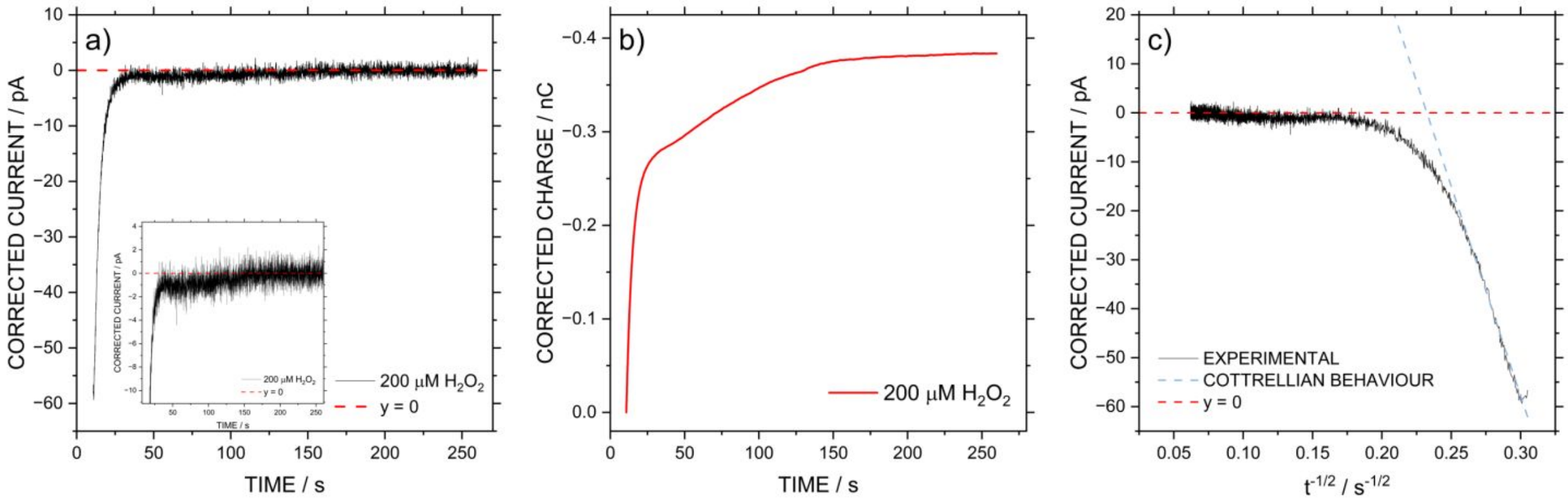

Figure S8: **a)** Current curve corrected from the blank extrapolated from the chronoamperometric measurement performed at 200 µM of H_2O_2, pH 4.3, plotted against time; **b)** integrated corrected charge from the chronoamperometry performed at 200 µM of hydrogen peroxide; **c)** associated Cottrell plot

Concerning the solution volume inside the CNP nanotip, in principle, it could be estimated from the integrated DPSC charge corrected by the capacitive contribution, applying the Faraday's law (Q = nFCV) and assuming the exhaustive conversion of the H_2O_2 aliquot. However, the main limitation of this approach is the lack of an independent comparative method to estimate the geometrical volume confined inside the CNP tip. To this end, only an apparent volume (V_{app}) could be estimated from the faradic charge associated with the H_2O_2 reduction. Considering the corrected charge values coming from the calibration curve reported in **Figure 6a,b** and assuming from the Faraday's law that n=2 (number of electrons involved in the PB/H_2O_2 redox reaction), F= 96485 C mol^{-1}, it is possible to estimate a V_{app} of 17.7 ± 1.3 pL for the associate experiment. This value is in the typical range expected for this kind of CNPs[4,5]. All the analytical parameters concerning the corrected charge (C), the bulk concentration and the apparent volume estimations are reported in **Table S1**.

Corrected Charge (C)	Bulk Concentration (µM)	Apparent Volume (V_{app}, pL)
3,90E-10	100	20.21
6,39E-10	200	16.56
1,00E-09	300	17.27
1,36E-09	400	17.62
1,69E-09	500	17.52
3,26E-09	1000	16.89

Table S1: Apparent confined volume (Vapp) calculated from the corrected charge according to Faraday's law (Q = nFCV) assuming exhaustive electrolysis of the confined H_2O_2 aliquot.

8. pH influence on the PB-NiHCF CNPs system

Figure S8 shows the CV profile of the same PB-Ni CNP sample under different stage of the experimental workflow previously described. Since the pH switches from 4.3 (red curve) to 7.4 (blue curve), a not negligible decrease in the PB domain is observed, while the NiHCF voltammetric signature maintain its profile almost unchanged. This behaviour could be attributable to the removal of the Ni-free PB amount in the CNP inner tip that, at neutral pH decrease its stability, according to

the literature. Thus, after the calibration stage, a decrease in both PB and NiHCF domains is recorded (green line), attributable to the high amount of hydrogen peroxide reduced at the 1000 μM concentration level following the calibration step. According to this point, it is necessary to note that the calibration stage consists of 43 DPSC runs due to the replicate measurements at different concentrations levels. However, despite the poor stability of the PB-NiHCF framework at relatively high concentration of hydrogen peroxide, the system shows excellent linearity up to 500 μM at pH 7.4, according to the R^2=0.996.

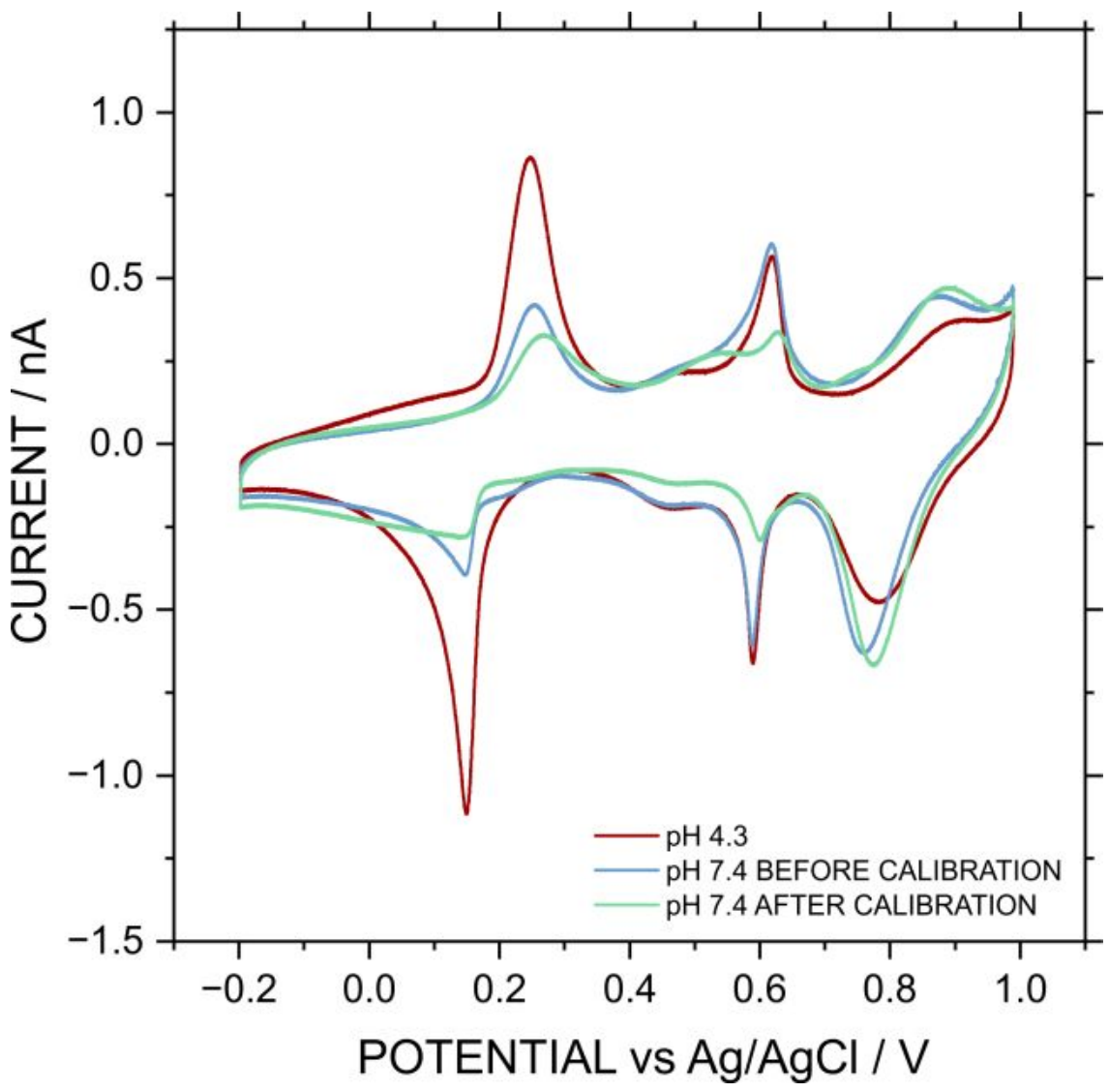

Figure S9: CV profiles of the PB-Ni CNP system recorded in the range +1.0V / -0.2V at 50 mV/s acquired at different experimental conditions: pH 4.3 (red line); pH 7.4 before the calibration (blue line); pH 7.4 after the calibration (green ine).

9. Reversibility and selectivity

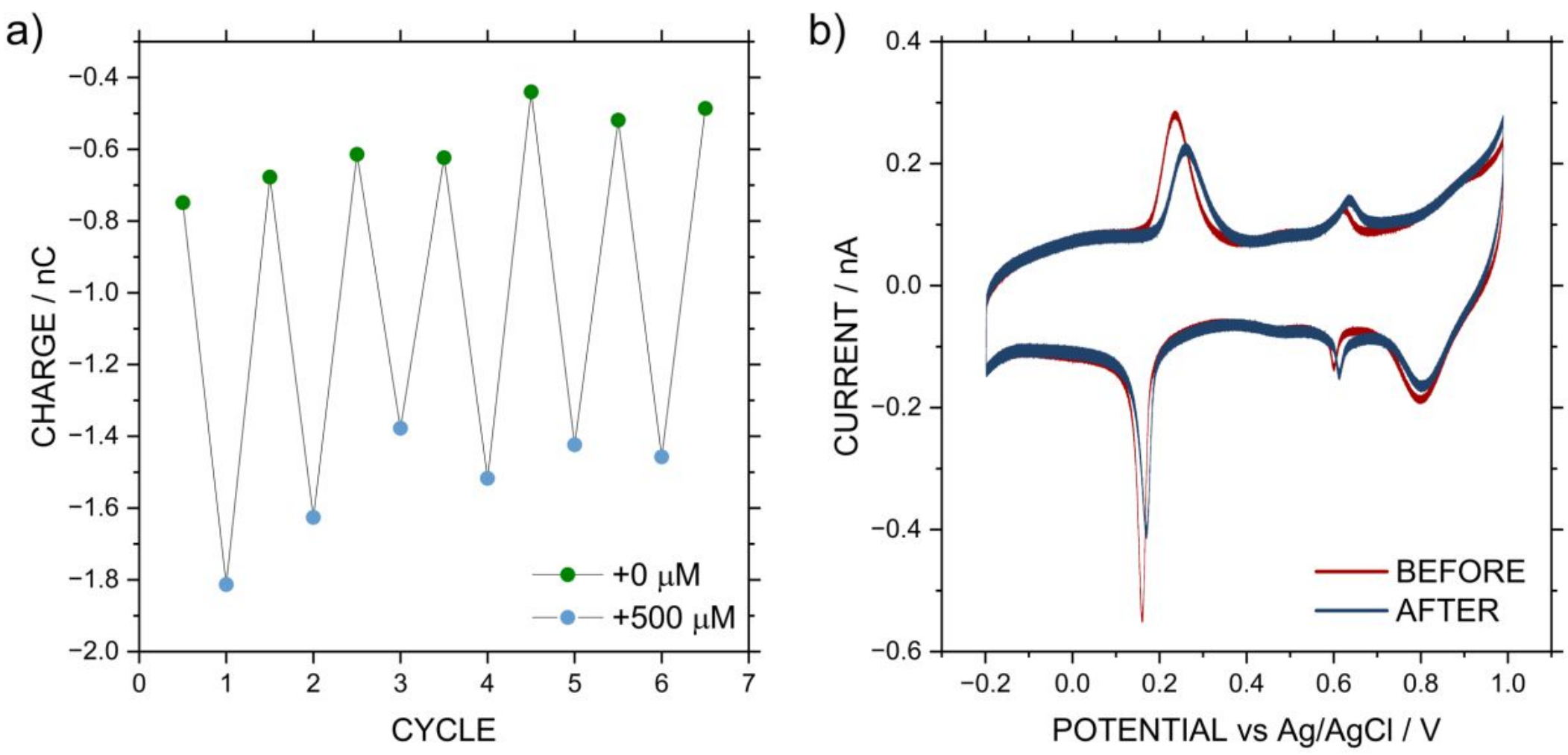

Figure S10: a) Charge values coming from DPSC measurements were the PB-Ni CNP device was cycled between 0 (green dots) and 500 (light blue dots) μM of H_2O_2; **b)** cyclic voltammograms before (red line) and after (blue line) the reversibility cycled experiment.

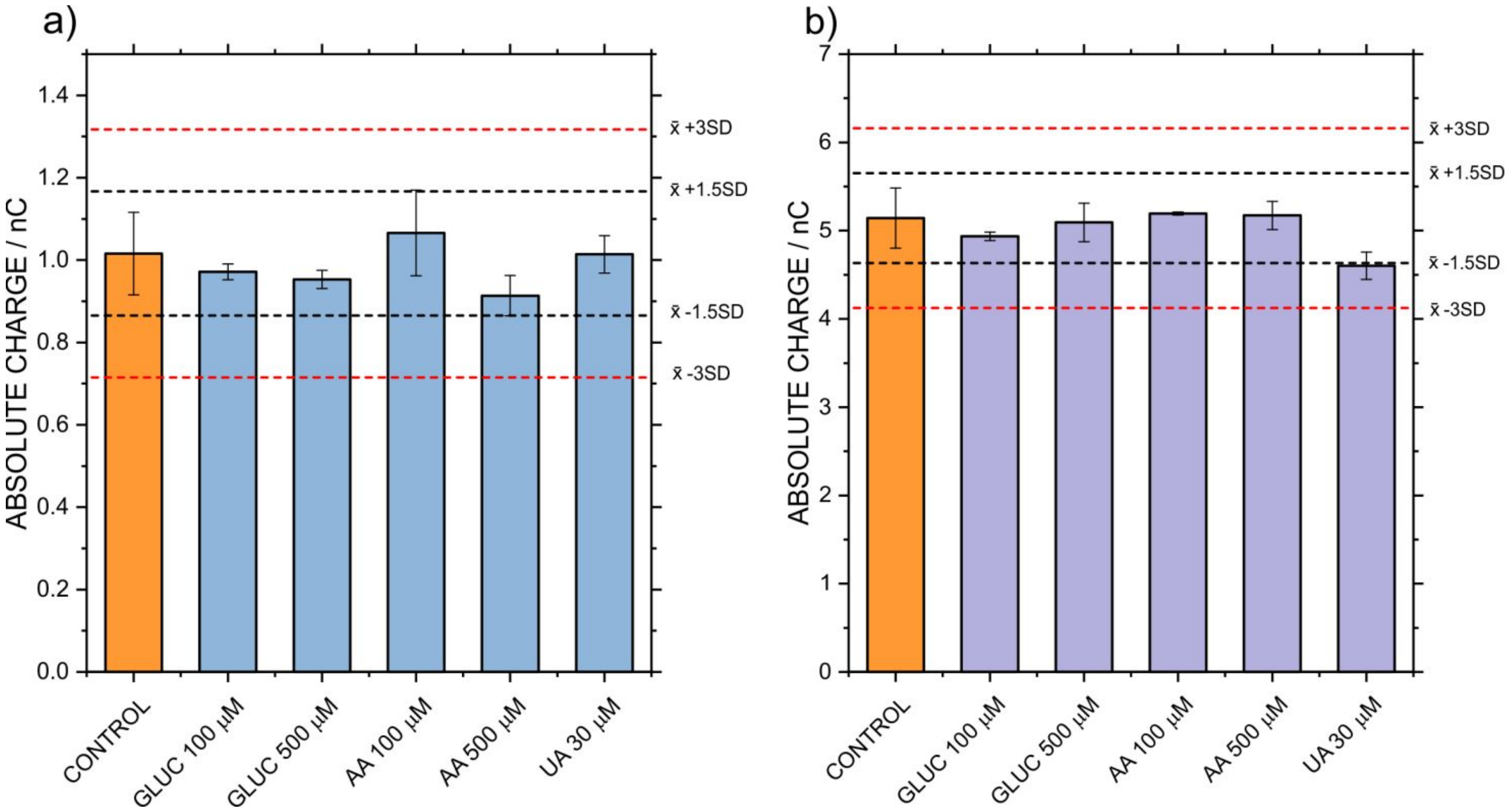

Figure S11: **a)** Absolute charge values coming from the amperometric response of the PB-Ni CNP system for 500 µM of H_2O_2, pH 4.3, in absence (control bar, orange column) and in the presence of D- (+)-Glucose (100 and 500 µM), L-Ascorbic Acid (100 and 500 µM) and Uric Acid (30 µM). The control limits were set at ±3s and ±1.5s of the mean value of three controls. Error bars represent the standard deviation coming from triplicate measurements; **b)** same selectivity experiment at pH 7.4.

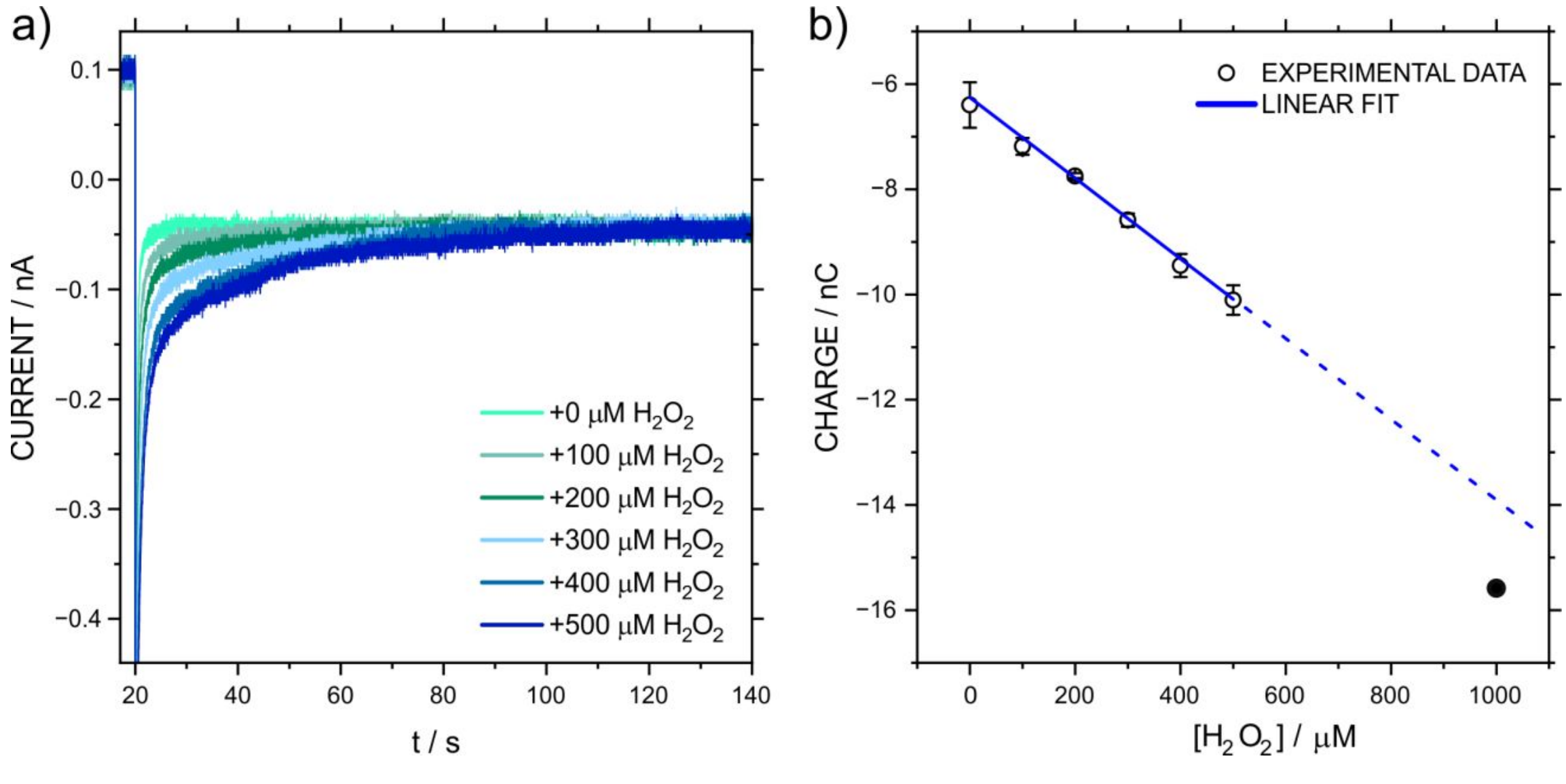

Figure S12: **a)** Double-potential step chronoamperometry responses related to the PB-Ni CNP tested at increasing H_2O_2 concentrations at pH 4.3. **b)** Corresponding calibration graph obtained from the charge values.

10. References

(1) Xue, S.; Li, B.; Yu, D.; Zhao, Y.; Wang, D. Prussian Blue Modified Carbon Nanopipette for Real-Time Voltametric Monitoring of K^+ in Single Living Cells. *Sensors Actuators B Chem.* **2026**, *462*, 140067.

(2) Ricci, F.; Palleschi, G. Sensor and Biosensor Preparation, Optimisation and Applications of Prussian Blue Modified Electrodes. *Biosens. Bioelectron.* **2005**, *21*, 3, 389–407.

(3) Molina-Osorio, A. F.; Wiorek, A.; Hussain, G.; Cuartero, M.; Crespo, G. A. Modelling

Electrochemical Modulation of Ion Release in Thin-Layer Samples. *J. Electroanal. Chem.* **2021**, *903*, 115851.

(4) Laucirica, G.; Carbonaro, A. B.; Nuzzo, M.; Crespo, G. A.; Cuartero, M. Scan-Rate-Induced Transition from Redox to Ion Current Rectification in Carbon Nanopipettes. *Anal. Chem.* **2026**, *98*, 18, 13197–13202.

(5) Laucirica, G.; Crespo, G. A.; Cuartero, M. Thin-Layer Behavior in Carbon Nanopipettes. Understanding the Iontronic-Electronic Contributions. *Anal. Chem.* **2025**, *97*, 32, 17659–17667.
